# Supplementary figures and images for: Structural and biochemical characterization of a novel thermophilic Coh01147 protease
Source: PLoS One. 2020 Jun 23;15(6):e0234958. doi: 10.1371/journal.pone.0234958 (PMC7310833; doi:10.1371/journal.pone.0234958)

## Slide 1
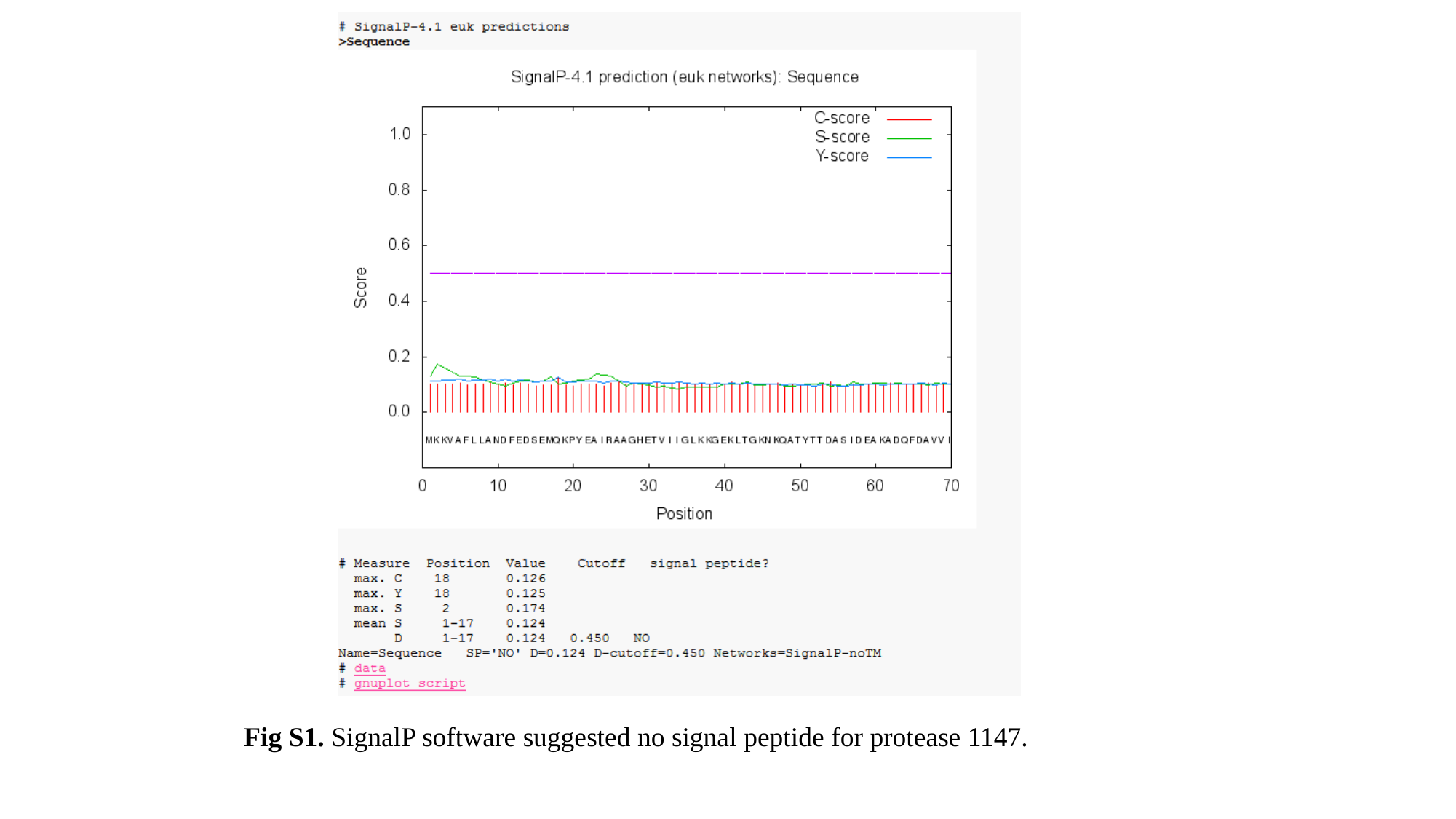

Fig S1. SignalP software suggested no signal peptide for protease 1147.

Supplement: S1 Fig — SignalP software suggested no signal peptide for protease 1147. (PPTX) [file pone.0234958.s001.pptx]

## Slide 1
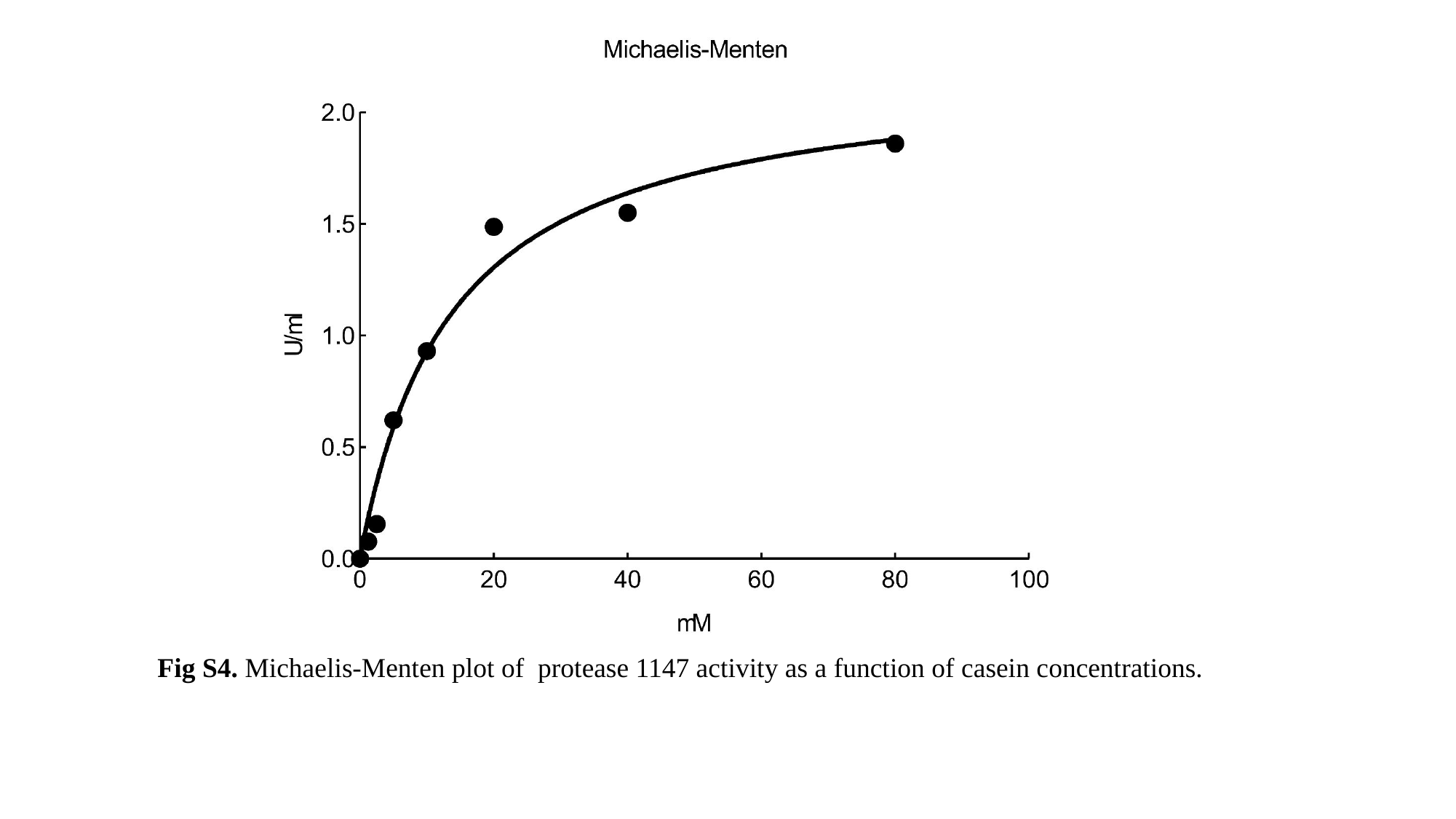

Fig S4. Michaelis-Menten plot of protease 1147 activity as a function of casein concentrations.

Supplement: S4 Fig — Michaelis-Menten plot of protease 1147 activity as a function of casein concentrations. (PPTX) [file pone.0234958.s004.pptx]
