# Supplementary material for: Structural and biochemical characterization of a novel thermophilic Coh01147 protease
Source: PLoS One. 2020 Jun 23;15(6):e0234958. doi: 10.1371/journal.pone.0234958 (PMC7310833; doi:10.1371/journal.pone.0234958)
Supplement: S2 Fig — ProSA local model quality/residue-wise energy plot shows most of the residues have negative energy. (PPTX) [file pone.0234958.s002.pptx]

## Slide 1
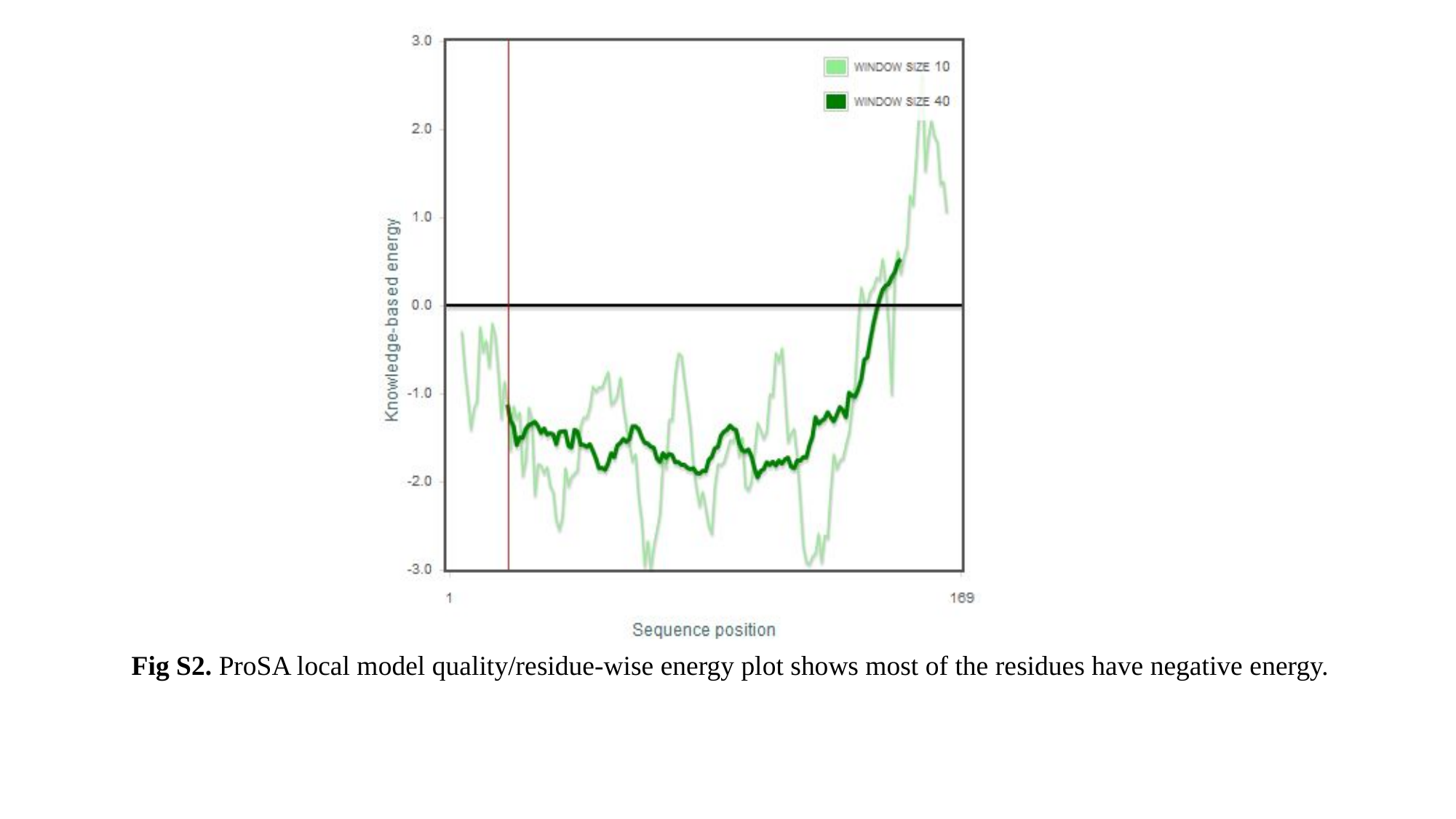

Fig S2. ProSA local model quality/residue-wise energy plot shows most of the residues have negative energy.
