# Supplementary material for: Structural and biochemical characterization of a novel thermophilic Coh01147 protease
Source: PLoS One. 2020 Jun 23;15(6):e0234958. doi: 10.1371/journal.pone.0234958 (PMC7310833; doi:10.1371/journal.pone.0234958)
Supplement: S3 Fig — M, DNA marker; 1, empty pET26b(+) vector; 2, recombinant pET26b(+) containing protease 1147 sequence; 3, double digestion of recombinant pET26b(+) by XhoI and NdeI restriction enzyme; 4 negative control PCR; 5, PCR from recombinant pET26b(+) vector with specific primers for protease 1147 sequence. (PPTX) [file pone.0234958.s003.pptx]

## Slide 1
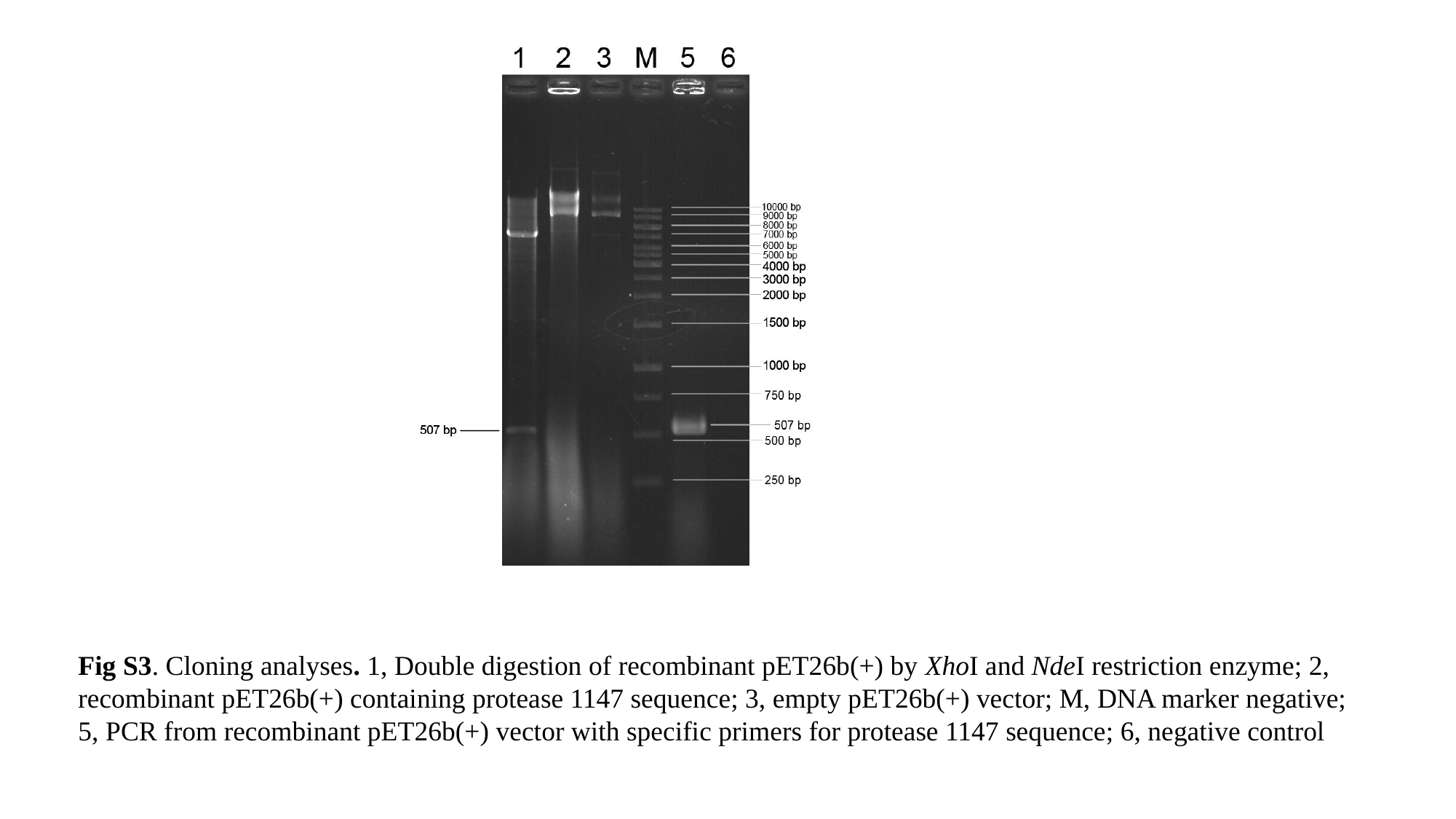

Fig S3. Cloning analyses. 1, Double digestion of recombinant pET26b(+) by XhoI and NdeI restriction enzyme; 2, recombinant pET26b(+) containing protease 1147 sequence; 3, empty pET26b(+) vector; M, DNA marker negative; 5, PCR from recombinant pET26b(+) vector with specific primers for protease 1147 sequence; 6, negative control
