# Supplementary material for: Structural and biochemical characterization of a novel thermophilic Coh01147 protease
Source: PLoS One. 2020 Jun 23;15(6):e0234958. doi: 10.1371/journal.pone.0234958 (PMC7310833; doi:10.1371/journal.pone.0234958)
Supplement: S1 File — Table A) Biochemical properties of protease 1147 and closely related proteases. NA, number of amino acids; MW, molecular weight; pI, theoretical isoelectric point; GRAVY, grand average of hydropathy; AI, aliphatic index; II, instability index. Table B) Effects of various metal ions on protease 1147 activity. Table C) The effect of metal ions, chemical compounds, inhibitors and surfactants on the activity of some other proteases. Table D) Type, Temperature, pH, substrate specificity and kinetic characterization of some other proteases. (DOCX) [file pone.0234958.s005.docx]

**Table A. Biochemical properties of protease 1147 and closely related proteases.** NA, number of amino acids; MW, molecular weight; pI, theoretical isoelectric point; GRAVY, grand average of hydropathy; AI, aliphatic index; II, instability index.

| **Protein** | **NA** | **MW** | **pI** | **GRAVY** | **AI** | **II** | **Organsism** |
| --- | --- | --- | --- | --- | --- | --- | --- |
| Protease 1147 | 169 | 18.8 | 5.9 | -0.37 | 86.1 | 35.3 | *Cohnella sp. A01 (This study)* |
| Protease I | 175 | 18.9 | 5.4 | -0.27 | 88.7 | 39 | [*A. Macrosporangiidus*](https://www.uniprot.org/uniprot/A0A1I7HDJ3) |
| Protease I | 175 | 19.1 | 5.7 | -0.37 | 87.5 | 36.4 | *Paenibacillus sp. 117* |
| ThiJ/PfpI protein | 177 | 19.8 | 5.2 | -0.36 | 82.8 | 46.3 | *Paenibacillus pasadenensis* |
| Protease YhbO | 179 | 19.6 | 5.3 | -0.42 | 82.9 | 37.7 | [*Paenibacillus sp. P22*](https://www.uniprot.org/uniprot/W8ZM78) |
| Protease PfpI | 168 | 18. | 4.9 | -0.24 | 95.9 | 37.6 | *Paenibacillus sp. HGF7* |
| Protease PfpI | 168 | 18.2 | 4.7 | -0.15 | 101.1 | 35.8 | *Paenibacillus sp. FSL R7-277* |
| Intracellular protease | 166 | 18.5 | 6.1 | -0.27 | 92.1 | 19.9 | [*Pyrococcus. horikoshii*](http://www.rcsb.org/pdb/explore/remediatedSequence.do?structureId=1G2I) |

**Table B**. Effects of various metal ions on protease 1147 activity.

| **Effector Molecule** | **Concentration** | **Relative activity (%)** |
| --- | --- | --- |
| **Metal ion** |  |  |
| Control | - | 100 |
| MgCl_2_ | 1mM  2mM  3mM | 104.54±5.9  106.97±9.8  98.48±1.4 |
| CaCl_2_ | 1mM  2mM  3mM | 105.15±6.7  115.45±1.8  98.18±0.9 |
| ZnCl_2_ | 1mM  2mM  3mM | 98.78±1.4  94.24±0.5  91.51±1.0 |
| AlCl_3_ | 1mM  2mM  3mM | 90.90±0.0  89.09±2.4  86.96±2.3 |
| NaCl | 1mM  2mM  3mM | 94.84±3.7  95.15±3.2  92.72±1.8 |
| KCl | 1mM  2mM  3mM | 98.48±1.4  99.69±0.5  96.66±1.9 |
| AgNO_3_ | 1mM  2mM  3mM | 99.69±0.5  98.48±1.4  90.30±4.5 |
| MnCl_2_ | 1mM  2mM  3mM | 97.27±0.9  96.06±0.5  93.63±1.8 |
| LiCl | 1mM  2mM  3mM | 97.87±2.3  96.36±4.8  91.81±0.9 |

**Table C.** The effect of metal ions, chemical compounds, inhibitors and surfactants on the activity of some other proteases.

| **Source** | **Cons.** | **Ca^2+^/**mM | **Na^2+^**/mM | **Zn^2+^**/mM | **Tween20**/% | **SDS**/% | **PMSF**/% | **EDTA/**mM | **E-64**/% | **Ref.** |
| --- | --- | --- | --- | --- | --- | --- | --- | --- | --- | --- |
| Protrase 1147 | 1 | 115.0 | 100 | 100 | 400.15 | 100 | 79.39 | 97.87 | 0.0 | In s. |
| Freesia reflacta | 1 | - | - | - | - | - | 83 | 85 | - | [1] |
| Vibrio harveyi | 1  5 | -  106 | -  - | -  85 | -  - | -  - | -  50 | -  50 | 100  69 | [2] |
| Ervatamia coronaria | 1 | - | - | - | - | - | 70 | 100 | - | [3] |
| Bacillus sp. SSR1 | 2  10 | -  177.5 | -  157.3 | -  83.4 | -  - | -  - | 14.79  - | 95.80  - | -  - | [4] |
| Bacillus sp. | 1  5 | 140  70 | -  - | 105  100 | -  - | -  - | -  10 | 90  - | -  - | [5] |
| B. mojavensis A21. | 1  5 | -  146.5 | -  100 | -  30.2 | -  - | 50  - | -  0 | -  59 | -  - | [6] |
| Horse gram | 10  100 | -  - | -  - | -  - | -  - | -  - | -  81 | 95  - | -  - | [7] |
| Babesia bigemina | 0.1  1 | -  - | -  - | -  - | -  - | -  - | -  70 | -  95 | 0  - | [8] |
| P. dicentrarchi | 0.1  1 | -  - | -  - | -  - | -  - | -  - | -  98 | -  95 | 2  - | [9] |
| Actinomycete | 100  250 | 450  - | -  450 | -  - | -  - | -  - | -  - | -  - | -  - | [10] |
| Bacillus sp. | 1  5 | -  140 | -  - | -  75 | -  - | 75  - | -  50 | -  100 | -  - | [11] |
| Litopenaeus vanamei | 1  5 | -  - | 93  85 | 4  1 | -  - | -  - | -  - | -  - | -  - | [12] |
| B. amyloliquefaciens SP1 | 1  5 | -  90.84 | -  100 | -  56.19 | -  - | 4.76  - | 90.48  80.95 | 96.18  90.48 | -  - | [13] |
| Bacillus invictae | 1  5 | -  127.18 | -  100.38 | -  101.88 | 100  83.08 | 19.48  - | -  0.0 | -  16.60 | -  - | [14] |
| Fluke Fasciola hepatica | 1  5 | -  - | -  - | -  - | -  - | -  - | -  96 | -  98 | -  0.0 | [15] |
| Cissus quadrangularis L. | - | - | - | - | - | - | - | - | - | [16] |
| Aeribacillus pallidus C10 | 1  5 | 115.3  120.6 | -  - | 83.11  114.8 | 20.0  37.67 | 79.33  116.7 | 69.54  0.0 | 79.92  100.4 | -  - | [17] |
| Vibrio sp. DA1-1 | 2  5 | 120.6  83.8 | 112.5  98.8 | 115.4  90.9 | 115.4  - | 64.1  56.1 | -  95 | -  40 | -  - | [18] |
| Bacillus mojavensis SA | 1  5 | -  156.62 | -  130.72 | -  32.93 | 83.98  - | 0.0  - | -  0.0 | -  16.61 | -  - | [19] |
| Fish waste | 1  5 | 110  - | 90  - | 50  - | -  - | 100  - | -  20 | -  100 | -  - | [20] |
| Aspergillus oryzae | 1 | 117.06 | - | 63.46 | - | - | 113.36 | 2.59 | - | [21] |
| Bacillus pumilus MP 27 | 0.1 | 690 | - | - | 700 | 0 | - | - | - | [22] |
| Bacillus cereus FT 1 | 0.1 | - | - | 10 | 180 | 80 | - | - | - | [23] |
| Bacillus caseinilyticus | 1  5  10 | -  82  - | -  30  - | -  78  - | 42  -  - | 55  -  - | -  0  - | -  96  - | -  -  - | [24] |
| Bacillus subtilis AKAL7 | 1  10 | -  125 | -  96 | -  25 | -  - | -  - | 23  - | 94  - | -  - | [25] |
| Exiguobacterium indicum AKAL11 | 1  10 | -  112 | -  85 | -  18 | -  - | -  - | 48  - | 85  - | -  - | [25] |
| Streptomyces Pseudogrisiolus NRC-15 |  | 150 (0.6% w/v) | 180 (0.6% w/v) | - | 200 (100 µmol/L) | - | - | - | - | [26] |
| Neocosmospora sp. N1 | 2  5  1  5 | 102  105  -  - | 68  70  -  - | 94  83  -  - | -  -  120  103 | -  -  73  51 | 5  0  -  - | 111  102  -  - | -  -  -  - | [27] |
| Virgibacillus sp. CD6 | 1  5 | -  85 | -  - | -  90 | 106  - | 77  - | -  69 | -  70 | -  - | [28] |
| Bacillus cereus PMW8 | 0.5  5 | -  110 | -  - | -  120 | 77  - | 160  - | -  100 | -  120 | -  - | [29] |
| Alkaline protease | 1  2.5  10 | -  -  1 | -  -  111 | -  -  140 | -  -  - | 90  -  - | -  -  - | -  -  - | -  -  - | [30] |
| Bacillus subtilis ATCC 6633 | 1  5 | 86  - | -  - | -  - | 106  - | 77  - | 51  - | -  102 | -  - | [31] |

**Table D.** Type, Temperature, pH, substrate specificity and kinetic characterization of some other proteases.

| **Source** | **Type** | **Temp** | **pH** | **Substrate** | **K_m_** | **V_max_** | **k_cat_ (s^-1^)** | **k_cat_ / K_m_** | **Ref.** |
| --- | --- | --- | --- | --- | --- | --- | --- | --- | --- |
| Protrase 1147 | Cysteine | 60 °C | 7 | Casein | 13.72 mM | 2.657 U/mg | 3.143×10^-3^ | 0.381M^-1^S^-1^ | In s. |
| Freesia reflacta | Cysteine | 30 °C | 6-7 | Casein | - | - | - | - | [1] |
| Vibrio harveyi | Cysteine | 50 °C | 8 | Casein | - | - | - | - | [2] |
| Ervatamia coronaria | Cysteine | 50 °C | 7.5-8 | Casein | - | - | - | - | [3] |
| Bacillus sp. SSR1 | Serine | 40 °C | 8-11 | Casein | - | - | - | - | [4] |
| Bacillus sp. | alkaline | 40 °C | 10-11 | Casein | 2 mg/ml**^-1^** | 289.8 µg min**^-1^** | - | - | [5] |
| B. mojavensis A21. | Serine | 60 °C | 8.5 | Casein | - | - | - | - | [6] |
| Horse gram | Cysteine | 40 °C | 5.5 | Azocasein | - | - | - | - | [7] |
| Babesia bigemina | Cysteine | 40 °C | 5.5 | Synthesized | 59.0 µg/ml**^-1^** | - | 0.306 | 5.19×10**^3^** | [8] |
| Bacillus subtilis BP-36 | alkaline | 70 °C | 9 | Casein | - | - | - | - | [32] |
| P. dicentrarchi | Cysteine | 40 °C | 5 | Synthesized | - | - | - | - | [9] |
| Actinomycete | alkaline | 70 °C | 9 | Casein | - | - | - | - | [10] |
| Bacillus sp. | Serine | 80 °C | 8 | Casein | - | - | - | - | [11] |
| Trichinella spiralis | Cysteine | 40 °C | 5.5 | Synthesized | 0.5091 μM/ml**^-1^** | 6.12 RFU/s μM | - | - | [33] |
| Litopenaeus vanamei | alkaline | 80 °C | 7 | Casein | 16.8 µM/ml**^-1^** | 82.6 µM/min | 38.2 | 2.27×10**^4^** | [12] |
| B. amyloliquefaciens SP1 | alkaline | 60 °C | 8 | Casein | 0.125 mg/ml**^-1^** | 12820 mg/ml**^-1^** | - | - | [13] |
| Bacillus invictae | Alkaline | 60 °C | 9-11 | Casein | - | - | - | - | [14] |
| Fluke Fasciola hepatica | Cysteine | 40 °C  40 °C | 5.5  7 | Casein  Synthesized | 160 µg/ml**^-1^**  54 µg/ml**^-1^** | -  - | 1.0  7.6 | 0.06×10**^2^**  1.4×10**^3^** | [15] |
| Cissus quadrangularis L. | Cysteine | 50 °C | 6 | Casein | - | - | - | - | [16] |
| Aeribacillus pallidus C10 | serine | 60 °C | 9 | Casein | 0.197 mg/ml | 7.29 lmol.ml**^-1^**.min**^-1^** | - | - | [17] |
| Vibrio sp. DA1-1 | alkaline | 55 °C | 10 | Casein | - | - | - | - | [18] |
| Bacillus mojavensis SA | - | 55 °C | 9 | Casein | - | - | - | - | [19] |
| Fish waste | - | 30 °C | 8 | Casein | 2 mg/ml**^-1^** | 25.0 Units/ml | 38.46 | 19.23 | [20] |
| Aspergillus oryzae | - | 55 °C | 8 | Casein | - | - | - | - | [21] |

1. Kaneda M, Yonezawa H, Uchikoba T. Purification and Characterization of a Cysteine Protease from Corms of Freesia, Freesia reflacta. Bioscience, Biotechnology, and Biochemistry. 1997;61(9):1554-9. doi: 10.1271/bbb.61.1554.

2. Liu P-C, Lee K-K, Tu C-C, Chen S-N. Purification and Characterization of a Cysteine Protease Produced by Pathogenic Luminous Vibrio harveyi. Current Microbiology. 1997;35(1):32-9. doi: 10.1007/s002849900207.

3. Sundd M, Kundu S, Pal GP, Medicherla JV. Purification and Characterization of a Highly Stable Cysteine Protease from the Latex of Ervatamia coronaria. Bioscience, Biotechnology, and Biochemistry. 1998;62(10):1947-55. doi: 10.1271/bbb.62.1947.

4. Singh J, Batra N, Sobti RC. Serine alkaline protease from a newly isolated Bacillus sp. SSR1. Process Biochemistry. 2001;36(8):781-5. doi: https://doi.org/10.1016/S0032-9592(00)00275-2.

5. Gupta A, Roy I, Patel RK, Singh SP, Khare SK, Gupta MN. One-step purification and characterization of an alkaline protease from haloalkaliphilic Bacillus sp. Journal of Chromatography A. 2005;1075(1):103-8. doi: https://doi.org/10.1016/j.chroma.2005.03.127.

6. Haddar A, Bougatef A, Agrebi R, Sellami-Kamoun A, Nasri M. A novel surfactant-stable alkaline serine-protease from a newly isolated Bacillus mojavensis A21. Purification and characterization. Process Biochemistry. 2009;44(1):29-35. doi: 10.1016/j.procbio.2008.09.003.

7. Jinka R, Ramakrishna V, Rao SK, Rao RP. Purification and characterization of cysteine protease from germinating cotyledons of horse gram. BMC Biochem. 2009;10:28. Epub 2009/11/19. doi: 10.1186/1471-2091-10-28. PubMed PMID: 19919695; PubMed Central PMCID: PMCPMC2784799.

8. Martins TM, do Rosário VE, Domingos A. Expression and characterization of the Babesia bigemina cysteine protease BbiCPL1. Acta Tropica. 2012;121(1):1-5. doi: https://doi.org/10.1016/j.actatropica.2011.09.008.

9. Shin SP, Han SY, Han JE, Jun JW, Kim JH, Park SC. Expression and characterization of cathepsin L-like cysteine protease from Philasterides dicentrarchi. Parasitology International. 2014;63(2):359-65. doi: https://doi.org/10.1016/j.parint.2013.12.007.

10. Gohel SD, Singh SP. Thermodynamics of a Ca(2+)-dependent highly thermostable alkaline protease from a haloalkliphilic actinomycete. Int J Biol Macromol. 2015;72:421-9. Epub 2014/08/26. doi: 10.1016/j.ijbiomac.2014.08.008. PubMed PMID: 25150113.

11. Kamran A, Ur Rehman H, Ul Qader SA, Baloch AH, Kamal M. Purification and characterization of thiol dependent, oxidation-stable serine alkaline protease from thermophilic Bacillus sp. Journal of Genetic Engineering and Biotechnology. 2015;13(1):59-64. doi: https://doi.org/10.1016/j.jgeb.2015.01.002.

12. Dadshahi Z, Homaei A, Zeinali F, Sajedi RH, Khajeh K. Extraction and purification of a highly thermostable alkaline caseinolytic protease from wastes Penaeus vannamei suitable for food and detergent industries. Food Chemistry. 2016;202:110-5. doi: https://doi.org/10.1016/j.foodchem.2016.01.104.

13. Guleria S, Walia A, Chauhan A, Shirkot CK. Purification and characterization of detergent stable alkaline protease from Bacillus amyloliquefaciens SP1 isolated from apple rhizosphere. Journal of Basic Microbiology. 2016;56(2):138-52. doi: 10.1002/jobm.201500341.

14. Hammami A, Hamdi M, Abdelhedi O, Jridi M, Nasri M, Bayoudh A. Surfactant- and oxidant-stable alkaline proteases from Bacillus invictae: Characterization and potential applications in chitin extraction and as a detergent additive. Int J Biol Macromol. 2017;96:272-81. Epub 2016/12/19. doi: 10.1016/j.ijbiomac.2016.12.035. PubMed PMID: 27988295.

15. Hemici A, Benerbaiha RS, Bendjeddou D. Purification and biochemical characterization of a 22-kDa stable cysteine- like protease from the excretory-secretory product of the liver fluke Fasciola hepatica by using conventional techniques. Journal of Chromatography B. 2017;1068-1069:268-76. doi: https://doi.org/10.1016/j.jchromb.2017.10.049.

16. Muthu S, Gopal VB, Karthik SN, Sivaji P, Malairaj S, Lakshmikanthan M, et al. Antibacterial cysteine protease from Cissus quadrangularis L. Int J Biol Macromol. 2017;103:878-88. Epub 2017/05/26. doi: 10.1016/j.ijbiomac.2017.05.107. PubMed PMID: 28539266.

17. Yildirim V, Baltaci MO, Ozgencli I, Sisecioglu M, Adiguzel A, Adiguzel G. Purification and biochemical characterization of a novel thermostable serine alkaline protease from Aeribacillus pallidus C10: a potential additive for detergents. Journal of Enzyme Inhibition and Medicinal Chemistry. 2017;32(1):468-77. doi: 10.1080/14756366.2016.1261131.

18. Chen X, Zhou C, Xue Y, Shi J, Ma Y. Cloning, expression, and characterization of an alkaline protease, AprV, from Vibrio sp. DA1-1. Bioprocess and Biosystems Engineering. 2018;41(10):1437-47. doi: 10.1007/s00449-018-1972-6.

19. Hammami A, Fakhfakh N, Abdelhedi O, Nasri M, Bayoudh A. Proteolytic and amylolytic enzymes from a newly isolated Bacillus mojavensis SA: Characterization and applications as laundry detergent additive and in leather processing. Int J Biol Macromol. 2018;108:56-68. Epub 2017/11/29. doi: 10.1016/j.ijbiomac.2017.11.148. PubMed PMID: 29180048.

20. R S, J J, A TS. Purification, characterization, molecular modeling and docking study of fish waste protease. International Journal of Biological Macromolecules. 2018;118:569-83. doi: https://doi.org/10.1016/j.ijbiomac.2018.06.119.

21. Ma X, Liu Y, Li Q, Liu L, Yi L, Ma L, et al. Expression, purification and identification of a thermolysin-like protease, neutral protease I, from Aspergillus oryzae with the Pichia pastoris expression system. Protein expression and purification. 2016;128:52-9. doi: 10.1016/j.pep.2016.08.008. PubMed PMID: 27539551.

22. Baweja M, Tiwari R, Singh PK, Nain L, Shukla P. An Alkaline Protease from Bacillus pumilus MP 27: Functional Analysis of Its Binding Model toward Its Applications As Detergent Additive. Frontiers in Microbiology. 2016;7(1195). doi: 10.3389/fmicb.2016.01195.

23. Asha B PM. Optimization of alkaline protease production by Bacillus cereus FT 1 isolated from soil. J App Pharm Sci. 2018;8(02):119-27. doi: 10.7324/JAPS.2018.8219.

24. Mothe T, Sultanpuram VR. Production, purification and characterization of a thermotolerant alkaline serine protease from a novel species Bacillus caseinilyticus. 3 Biotech. 2016;6(1):53-. Epub 02/13. doi: 10.1007/s13205-016-0377-y. PubMed PMID: 28330122.

25. Hakim A, Bhuiyan FR, Iqbal A, Emon TH, Ahmed J, Azad AK. Production and partial characterization of dehairing alkaline protease from Bacillus subtilis AKAL7 and Exiguobacterium indicum AKAL11 by using organic municipal solid wastes. Heliyon. 2018;4(6):e00646-e. doi: 10.1016/j.heliyon.2018.e00646. PubMed PMID: 30009270.

26. Mostafa E-SE, Saad MM, Awad HM, Selim MH, Hassan HM. Optimization Conditions of Extracellular Proteases Production from a Newly Isolated Streptomyces Pseudogrisiolus NRC-15. E-Journal of Chemistry. 2012;9(2). doi: 10.1155/2012/168540.

27. Matkawala F, Nighojkar S, Kumar A, Nighojkar A. A novel thiol-dependent serine protease from Neocosmospora sp. N1. Heliyon. 2019;5(8):e02246. doi: 10.1016/j.heliyon.2019.e02246.

28. Lam MQ, Nik Mut NN, Thevarajoo S, Chen SJ, Selvaratnam C, Hussin H, et al. Characterization of detergent compatible protease from halophilic Virgibacillus sp. CD6. 3 Biotech. 2018;8(2):104-. Epub 01/31. doi: 10.1007/s13205-018-1133-2. PubMed PMID: 29404232.

29. Esakkiraj P, Meleppat B, Lakra AK, Ayyanna R, Arul V. Cloning, expression, characterization and application of protease produced by Bacillus cereus PMW8. RSC Advances. 2016;6(45):38611-6. doi: 10.1039/C5RA27671C.

30. Ramkumar A, Sivakumar N, Gujarathi AM, Victor R. Production of thermotolerant, detergent stable alkaline protease using the gut waste of Sardinella longiceps as a substrate: Optimization and characterization. Scientific Reports. 2018;8(1):12442. doi: 10.1038/s41598-018-30155-9.

31. Chatterjee J, Giri S, Maity S, Sinha A, Ranjan A, Rajshekhar, et al. Production and characterization of thermostable alkaline protease of Bacillus subtilis (ATCC 6633) from optimized solid-state fermentation. Biotechnology and Applied Biochemistry. 2015;62(5):709-18. doi: 10.1002/bab.1309.

32. Mashayekhi Mazar F, Shahbaz Mohammadi H, Ebrahimi-Rad M, Gregorian A, Omidinia E. Isolation, Purification and Characterization of a Thermophilic Alkaline Protease from Bacillus subtilis BP-36. Journal of Sciences, Islamic Republic of Iran. 2012;23(1):7-13.

33. Qu Z-g, Ma X-t, Li W-h, Zhang N-z, Yue L, Cui J-m, et al. Molecular characterization of a cathepsin F-like protease in Trichinella spiralis. Parasites & Vectors. 2015;8(1):652. doi: 10.1186/s13071-015-1270-y.
