# Supplementary material for: Structural and biochemical characterization of a novel thermophilic Coh01147 protease
Source: PLoS One. 2020 Jun 23;15(6):e0234958. doi: 10.1371/journal.pone.0234958 (PMC7310833; doi:10.1371/journal.pone.0234958)
Supplement: S1 Raw images — (PDF) [file pone.0234958.s006.pdf]

Raw-cloning analyses

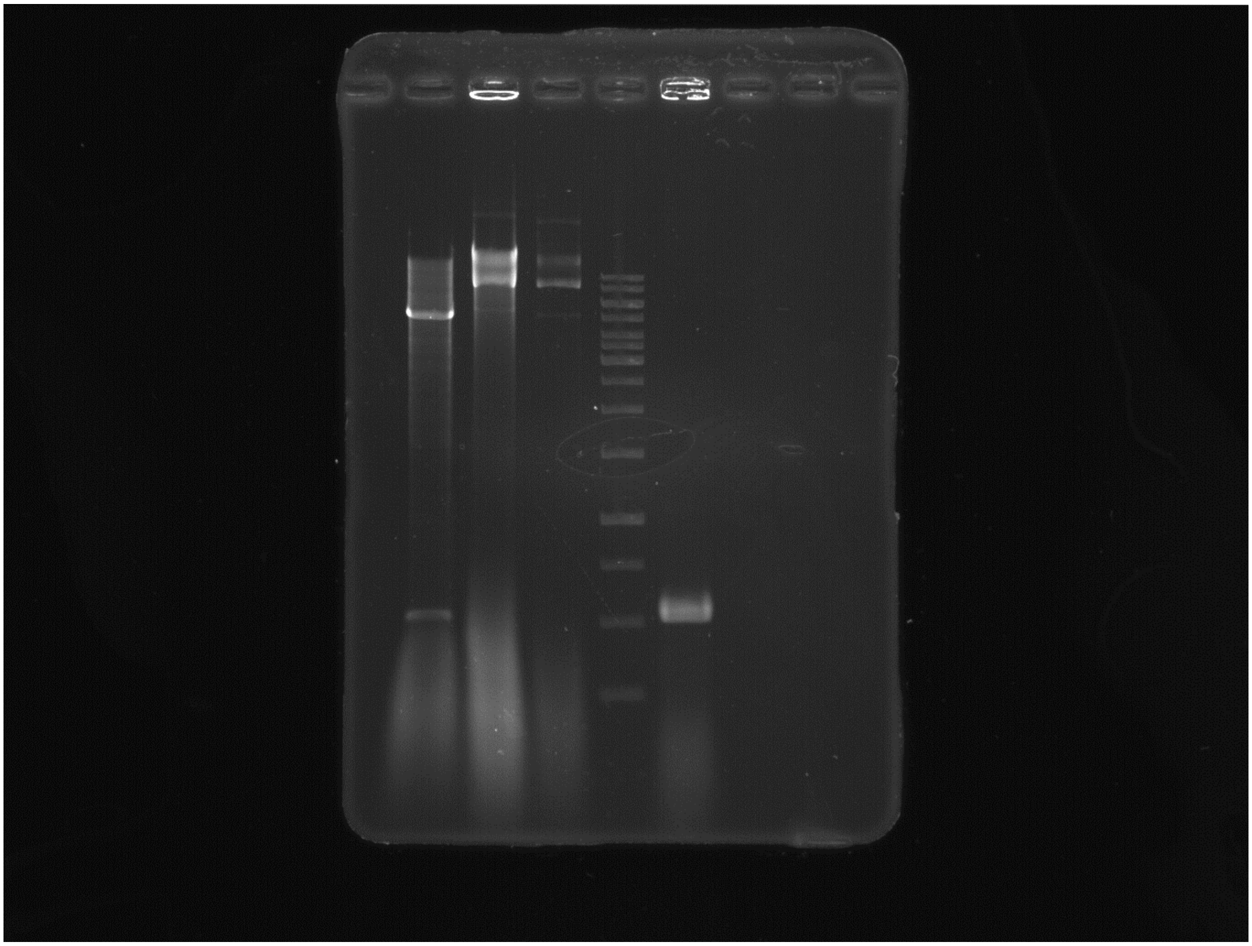

Raw-cloning analyses with caption

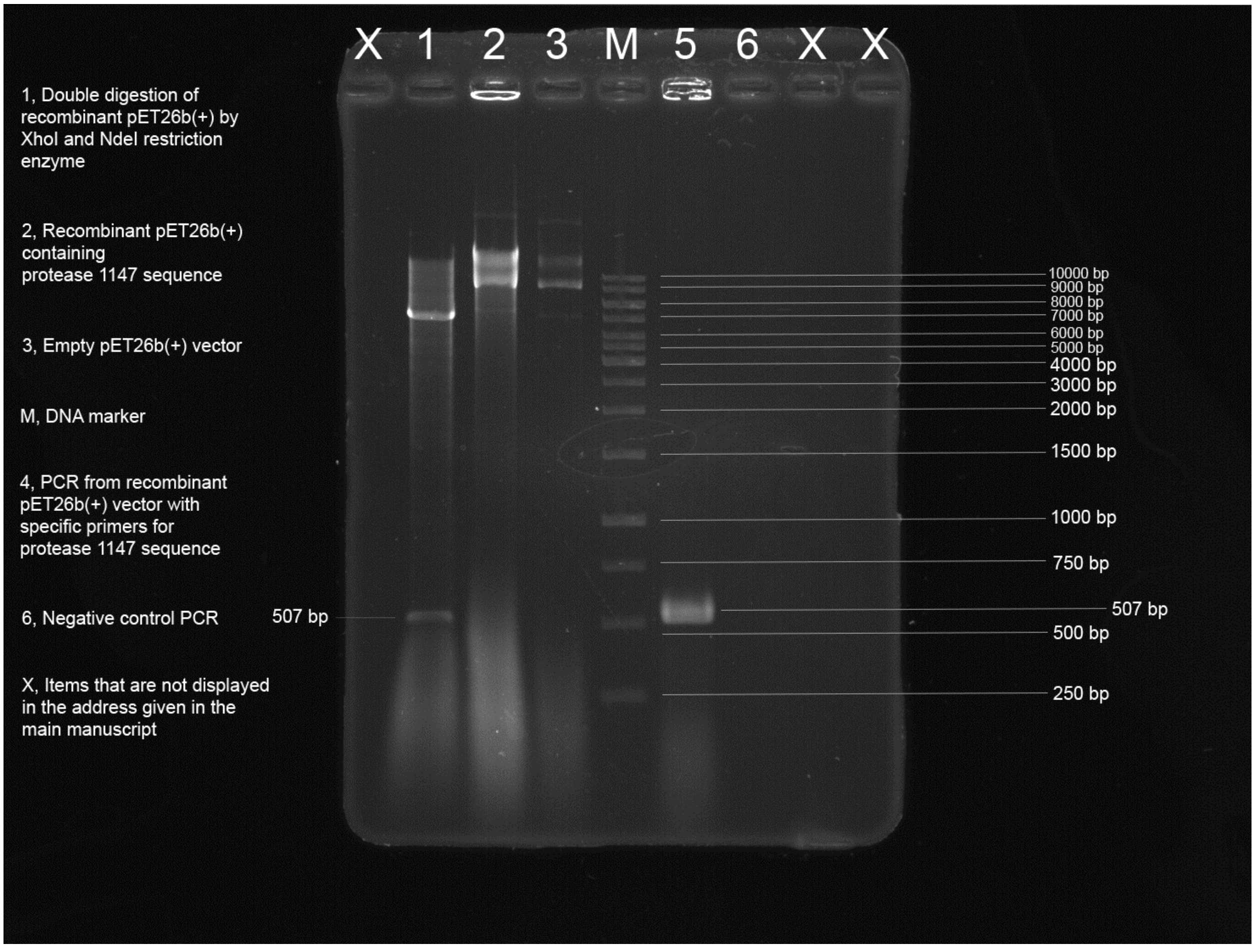

The figure of the address given in the main manuscript.

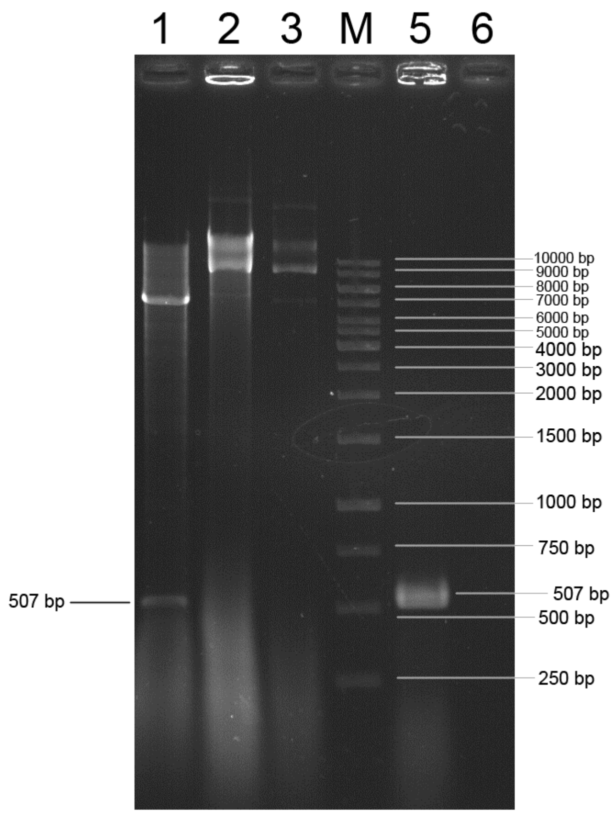

Raw-SDS-PAGE analyses

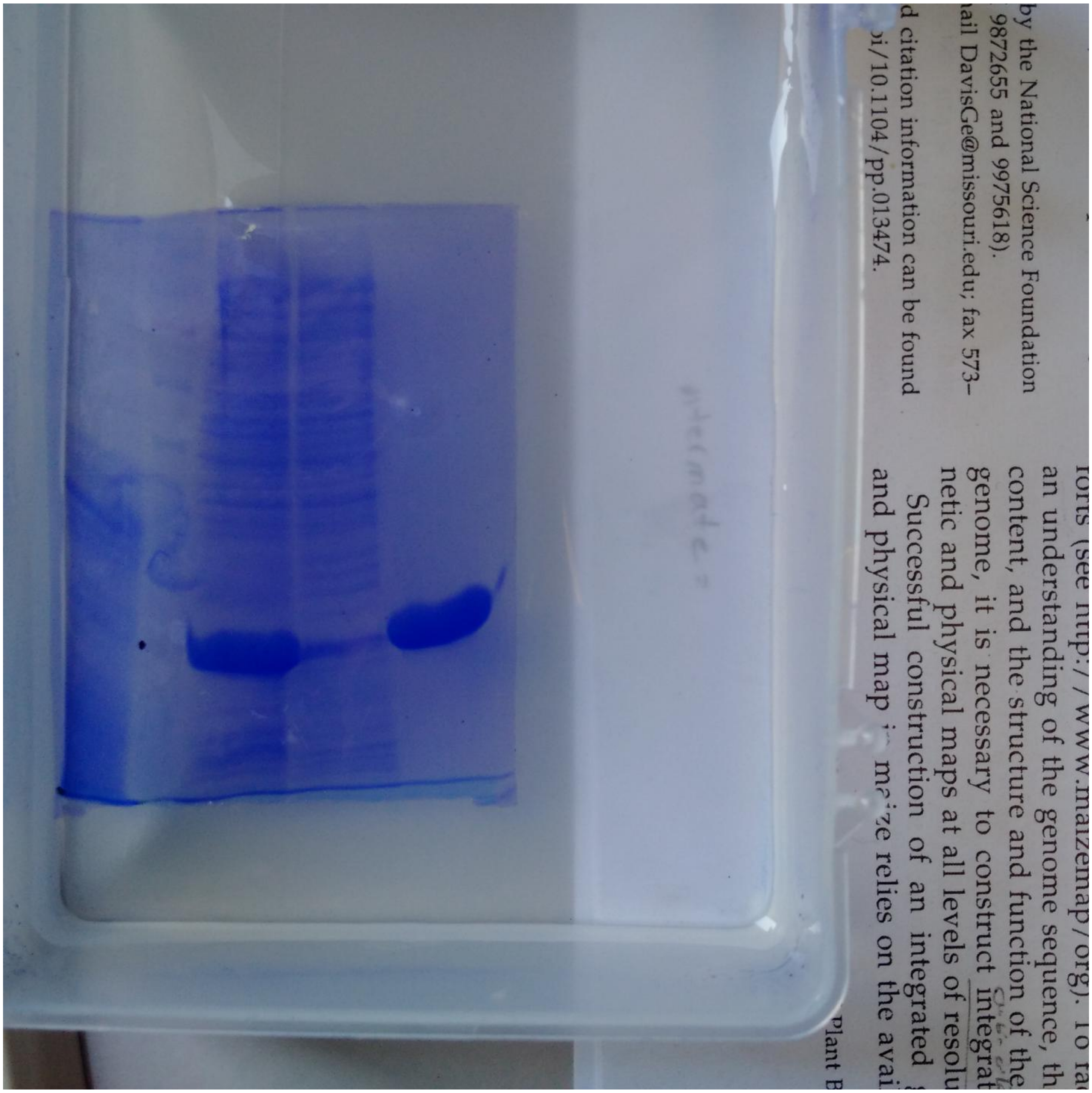

Raw-SDS-PAGE analyses with caption

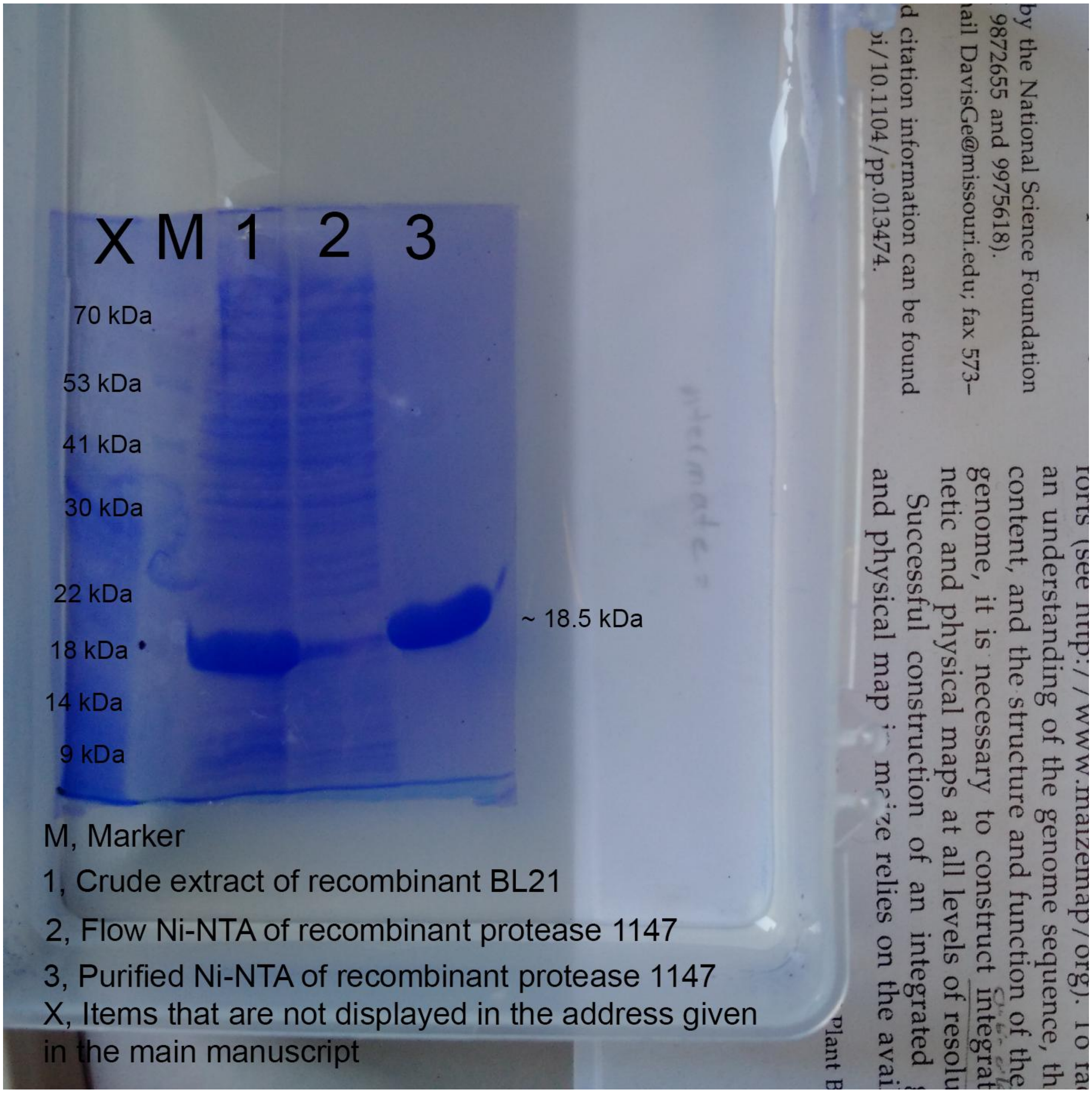

M, Marker

1, Crude extract of recombinant BL21

2, Flow Ni-NTA of recombinant protease 1147

3, Purified Ni-NTA of recombinant protease 1147

X, Items that are not displayed in the address given in the main manuscript

The **figure 8A** of the address given in the main manuscript.

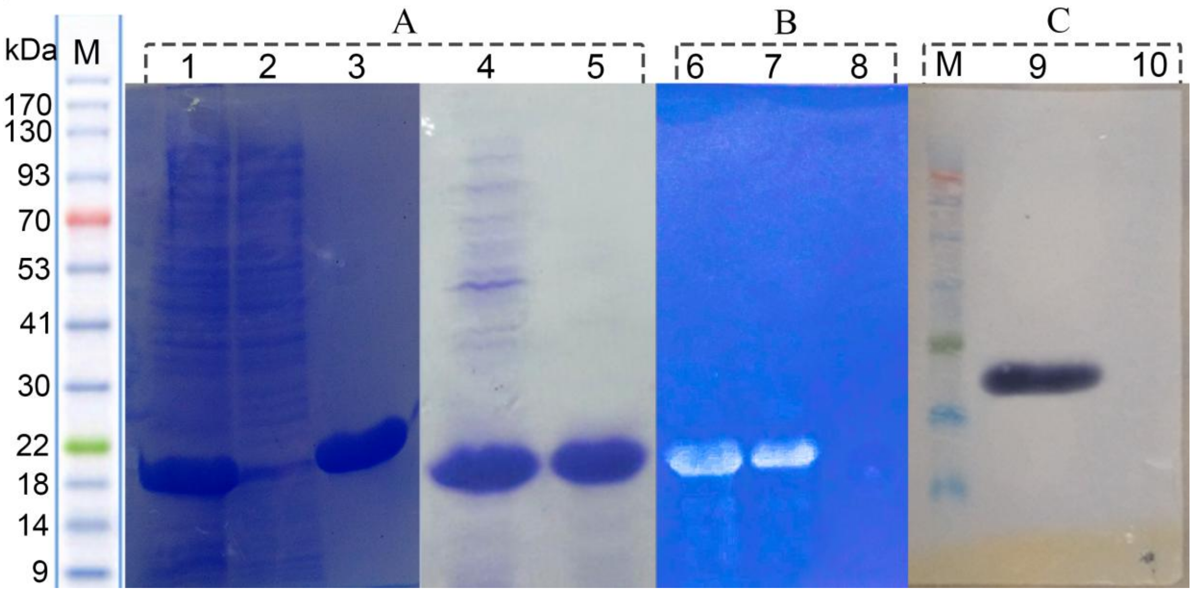

Fig 8. SDS-PAGE, western blot and zymogram analysis of recombinant protease 1147*. (A) SDS-PAGE analysis of recombinant protease 1147. Lane 1) crude extract of recombinant BL21, lane2) crude extract of non-recombinant BL21 (negative control), lane 3) Ni-NTA purified.*

Raw-SDS-PAGE heat shock purified analyses

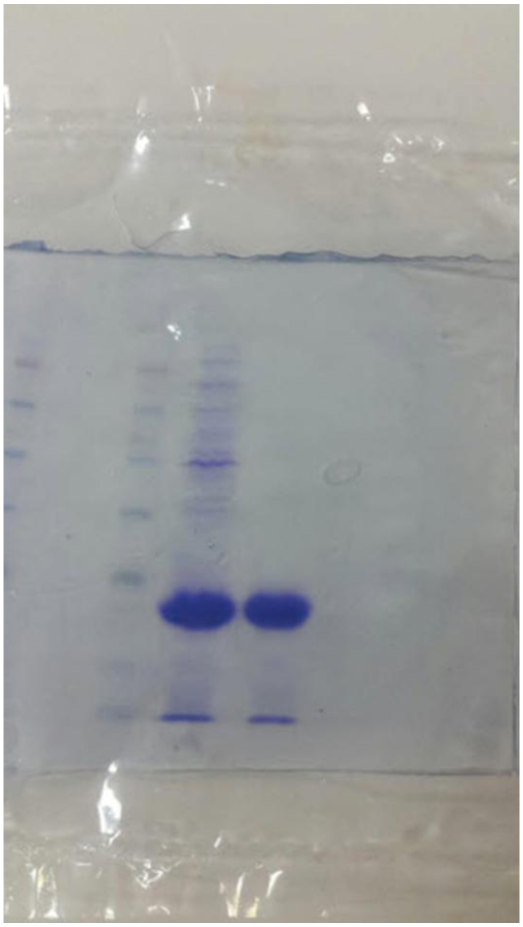

Raw-SDS-PAGE heat shock purified analyses with caption

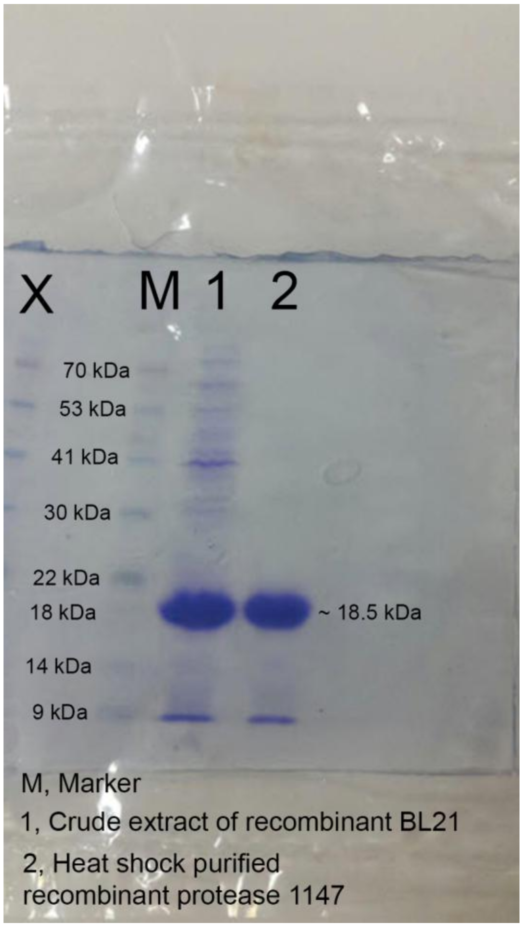

The **figure 8A** of the address given in the main manuscript.

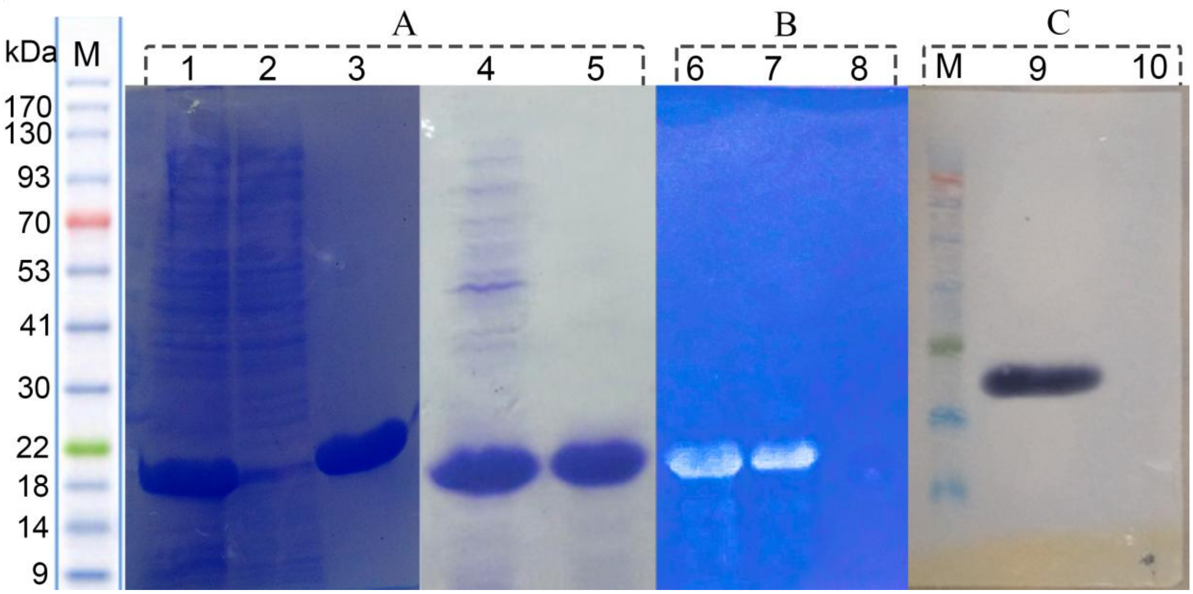

Fig 8. SDS-PAGE, western blot and zymogram analysis of recombinant protease 1147. *(A) SDS-PAGE analysis of recombinant protease 1147. Lane 4) crude extract of recombinant BL21 and lane 5) heat shock purified recombinant protease 1147.*

Raw-NATIVE-PAGE zymogram analyses

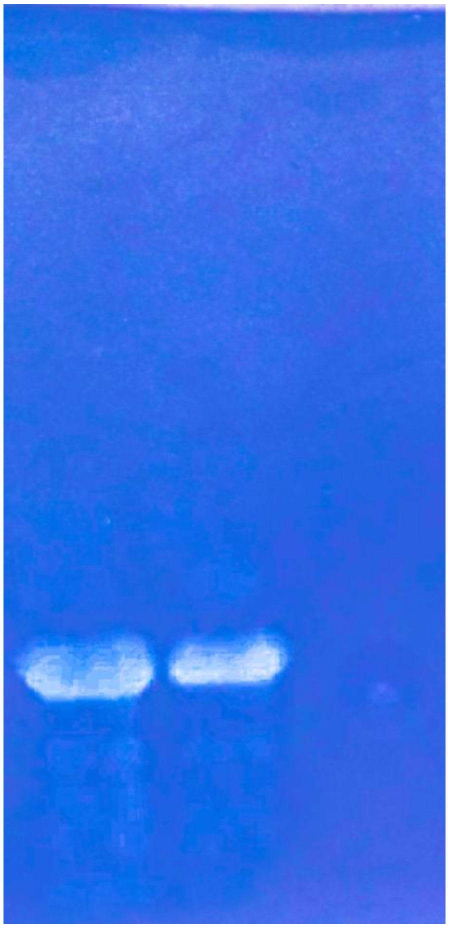

Raw-NATIVE-PAGE zymogram analyses with caption

1, Ni-NTA purified
Native-PAGE zymogram

2, Crude extract of
recombinant BL21
Native-PAGE zymogram

3, Crude extract of
non-recombinant BL21
(negative control)
Native-PAGE zymogram

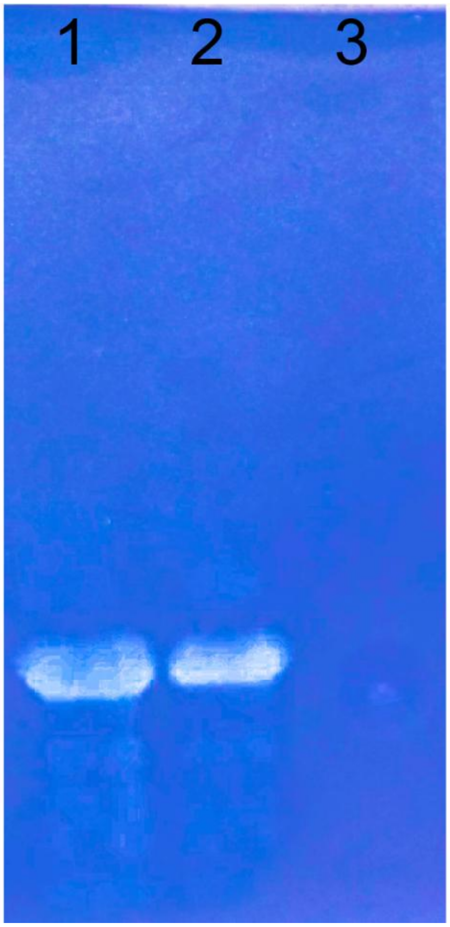

The **figure 8B** of the address given in the main manuscript.

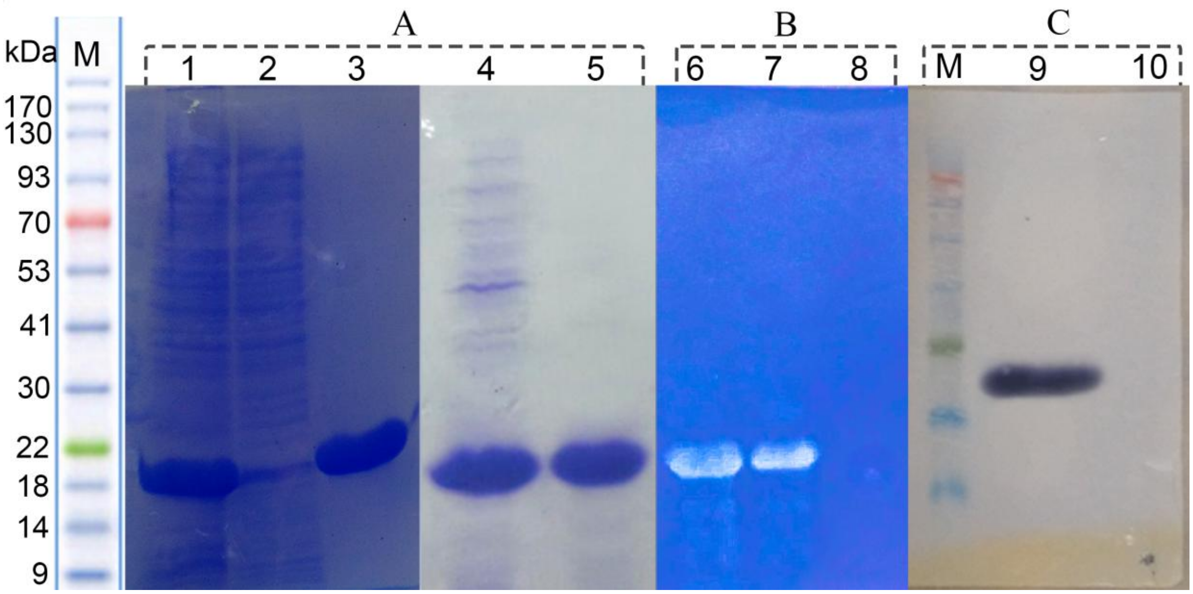

Fig 8. SDS-PAGE, western blot and zymogram analysis of recombinant protease 1147*. (B) Native-PAGE zymogram analysis of 6) Ni-NTA affinity column and, 7) heat shock single-step method purified protease 1147, 8) crude extract of non-recombinant BL21 (negative control).*

Raw-Western blot analyses

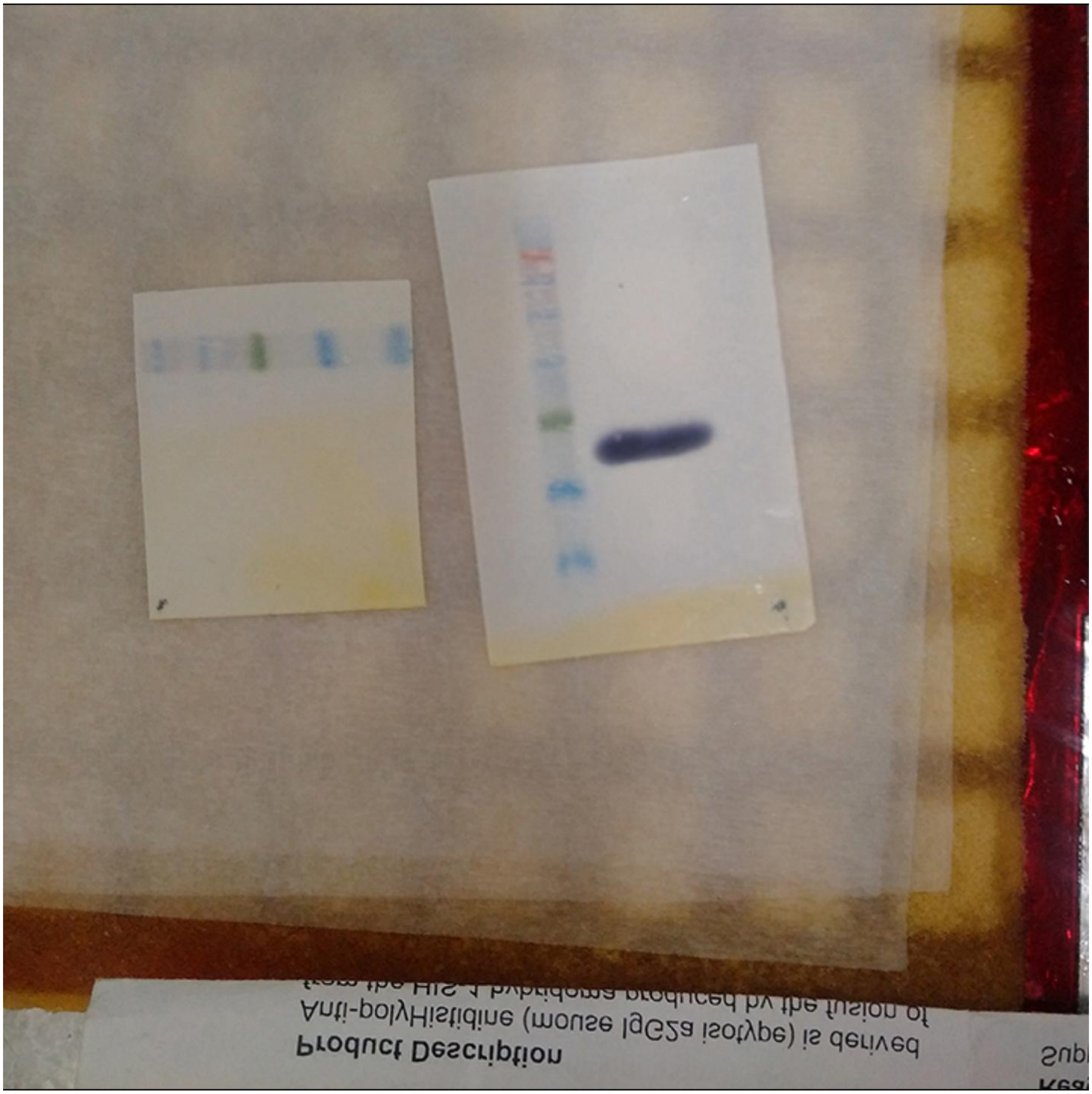

Raw-Western blot analyses with caption

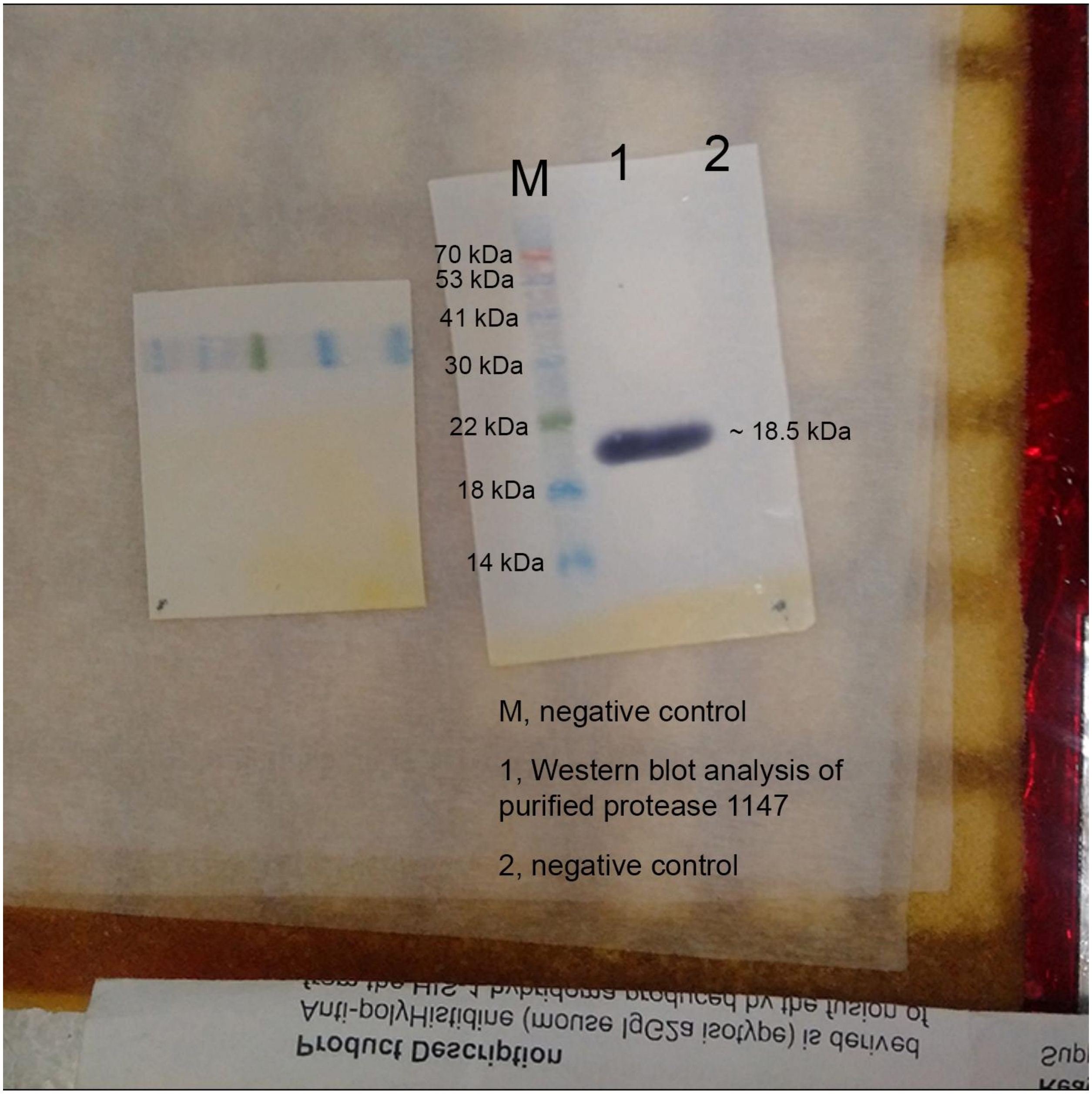

The **figure 8C** of the address given in the main manuscript.

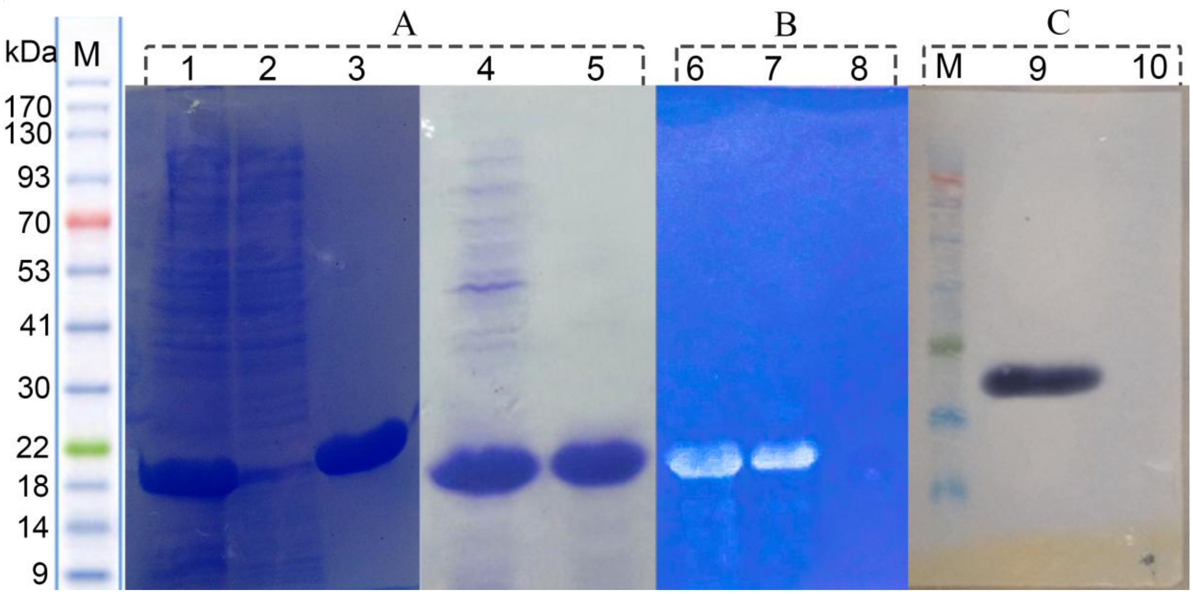

Fig 8. SDS-PAGE, western blot and zymogram analysis of recombinant protease 1147*. (C) Western blot analysis of (9) Ni-NTA affinity column purified protease 1147, and (10) negative control.*
