# Supplementary material for: Structural and biochemical characterization of a novel thermophilic Coh01147 protease
Source: PLoS One. 2020 Jun 23;15(6):e0234958. doi: 10.1371/journal.pone.0234958 (PMC7310833; doi:10.1371/journal.pone.0234958)
Supplement: Attachment — Submitted filename: Response to Reviewers.docx [file pone.0234958.s007.docx]

**Journal:**	*PLOS ONE*

**Manuscript #:**	*PONE-D-19-30576*

**Title of Paper:** *Structural and Biochemical Characterization of a Novel Thermophilic*

*Coh01147 Protease*

**Authors:**	*Hossein Tarrahimofrad, Amir Meimandipour, Sareh Arjmand, Mohammadtaghi Beigi Nassiri, Ehsan Jahangirian, Hossein Tavana, Javad Zamani, Somayyeh Rahimnahal, Saeed Aminzadeh*

**Dear Dr. Adam Lesner**

We appreciate the time and efforts by the editor and referees in reviewing this manuscript. We have addressed all issues indicated in the review report carefully and believed that the revised version can meet the journal publication requirements.

**Yours sincerely**

**Saeed Aminzadeh**

**Reviewer #1:**

**Major:**
**1.** May I suggest to modify all the subtitles in Result section to be more “result – orientated”. In other words, not on what the experiments have been done, but what the results obtained.
*We appreciate the Reviewer’s careful reading of the manuscript. The subtitles in the Results section were modified to be more “result-oriented”. The modified subtitles were written in blue font.*

**2. Fig 5,** For the MD simulation and docking results, can authors comment on how repeatable/robust they are? In other words, will docking/MD generate other simulations if run multiple times?

*To ensure that the results are repeatable, Docking, and MD simulation were repeated three times. For this purpose, the ligands and the modeled protease 1147 were prepared. Using Molegro Virtual Docker (MVD) software, protein-ligand dockings were performed between protease 1147 and SDS as well as protease 1147 and Tween20. The molecular dynamics simulation were repeated as well. All dynamic molecular factors such as temperature and pressure adjustment as well as sample topology files were performed according to the initial run. Repeating the experiments indicated that the results are reproducible.*

*Performing the experiments in triplicate was noted in the materials and methods, and footnote of figures 6 and 7 (in red font), and figure 7 was updated.*

**Minors:**
**3. Abstract:** Kcat to kcat

*The word K_cat_ was replaced with k_cat_.*

**4. Page 12, line 7,** what is the “TCA solution”?

*TCA stands for Trichloroacetic acid*. *The abbreviation was introduced in the manuscript.*

**5. Table 1,** isoelectric point is pI not Pi.

*The typo was corrected, and table 1 moved to supplementary file.*

**6. Page 19, line 7,** please spell out RMSD.

*It was spelled out in the materials and methods, section; homology modeling and validation.*

**7. Fig 7b,** y-axis label is missing

*The y-axis label was added in the RMSD graph.*

**8. Fig 6,** May consider moving it to SI since less significant

*We feel this image can help in the proper retrieval of data because it shows the loss of connectivity between the 1147-SDS complex at the end of the MD. Because, as explained in the manuscript, at the beginning of molecular dynamics simulation, SDS establishes a hydrogen bond with the protein. This hydrogen bond was established with the amino acid Lysine 19 of the protein shown in Figure 6 (a and b). However, at the end of the molecular dynamics simulation, the hydrogen bond between SDS and the amino acid lysine 19 is lost, as shown in part c and d of Figure 6. In Overall, Figure 6 shows the loss of the hydrogen bond between SDS and protease 1147. In Compared with Figure 5, which shows the maintenance and enhancement of hydrogen bonding, Fig 6 can show the difference in the interaction at the beginning and end of the molecular dynamics simulation between the protein and the ligand.*

**9. Tables 2 and 3,** the significant digits – two digits should be enough. And no way to achieve 4 digits there.

*Thanks for the comment. The digits were corrected.*

**Reviewer #2:**

Aminzadeh and coworkers describe a structural and biochemical characterization of a novel protease from thermophilic Coh01147 bacteria (Cohnella sp.). The structural analysis involves sequence homology analysis to identify a related known structure, and then homology modeling to produce a model of the enzyme. Molecular dynamics simulations are used to suggest a specific complex formation with surfactant Tween 20. A web-based server is used to predict a number of biophysical parameters for the protein. The protease is cloned and expressed and purified using a single-step His-tag strategy. Enzyme activity is characterized using zymography, temperature-dependent activity measurements, and a Michaelis-Menten analysis using casein. Other fluorescence spectroscopy studies were reported, including ANS binding; temperature and pH effects upon fluorescence and CD were also reported. There are a number of criticisms of the report (in no particular order):

**1.** The authors report kcat as "Kcat", suggesting it is an equilibrium constant instead of the correct rate constant nomenclature

*Thanks for the comment. The word K_cat_ was replaced with k_cat_ in the text.*

**2.** In several instances the authors report 4-5 digits of precision for values. This level of precision would seem unsupported

*Thanks for the comment. The digints were corrected.*

**3.** The interaction with Tween20 appears somewhat inconsistent. The authors report molecular dynamics studies show specific Tween 20 binding, but can only provide a single H-bond for such interaction. They also report Tween20 increases enzyme activity, but then also report that unfolding is induced by addition of Tween20.

*Thanks for the comment. This is a very important question. MD results showed that the values of RMSD, RMSF, and Rg of the tween 20-1147 complex were much lower than that of protease 1147. Even the number of hydrogen bonds increased at the end of the molecular dynamics. These results confirm that the binding of tween 20 enhances the stability of the protein 1147 structure.*

*Several articles reported that heat-resistant proteins generally have a compact structure. This compact structure allows the protein to withstand temperature and pH changes and maintain its structure. This property is also transmitted to the active site of the enzyme, and the amino acids in the active site are located in a tight space in the protein structure. Tween 20 is a surfactant and can act as an oil and increase the flexibility of the protein structure. Tween 20 affects the active site of the enzyme, facilitating the opening of the active site and entry of the substrate. In fact, Tween 20 converts the compact structure of the protein into a flexible structure.*

*So far, there have been no reports of the mechanism of action of Tween 20 on protein, but in this study we concluded that Tween 20 can reduce protein structure compactness and makes the active site’s amino acids more readily linked to the substrate and increases the activity of the enzyme. This increase in activity is due to the fact that tween 20 changes the protein structure. This structural changes caused variations in the wavelength of the spectrophotometric studies.*

*Furthuremore, to ensure that the results are repeatable, Docking, and MD simulation were repeated three times and figure 7 was updated.*

**4.** Enzyme assays are potentially problematic for proteases that might self-digest (thereby reducing enzyme concentration over time). The authors do not address this possibility.

*The protease used in this study came from a thermophilic source. In general, enzymes from thermophilic sources have a compact structure. Because hydrophobic amino acids make up the bulk of the protein. This helps to increase the protein's ability to withstand high temperatures and maintain structure against pH changes. This compact structure allows protease 1147 to endure against autolysis conditions. To ensure about the lack of self-digestion, we subjected the recombinant protease 1147 in three different times (one day, one week and two weeks after expression ) on the SDS-PAGE. The result indicated in figure 1R. According to this qualificative result, the autolysis can not be problametic in our experiments. We should add that we used the recombinant protease 1147 one day after expression and exactly after purification. Furthermore, all steps of purification were performed at at 4 °C or using a cold chain (ice).*

Figure R1. SDS-PAGE of recombinant protease 1147 1: One day after expression, 2: One week after expression, 3: Two week after expression. M:: Protein marker.

**5.** The Michaelis-Menten study utilizes casein as a substrate. There is likely more than one site of cleavage with such a large and complex polypeptide, but the MM treatment assumes a single substrate (i.e. site of cleavage). The authors may be fitting experimental data to an inappropriate model

*We understand the term MM treatment in your question, as Molecular modeling (MM). in this case molecular modeling (MM) treatment and in vitro studies have shown that enzyme 1147 acts as an active domain of protease function. Protein-protein docking was not performed. We did not do molecular modeling research for the casein as substrate.* *We just performed Tween20 and SDS as ligand for molecular modeling and did not performed substrate (Casein).*

**6.** In the purification the authors report a mass of imidazole in the elution buffer, but no concentration. It is therefore impossible to repeat the study with the data provided

*Page 10, Lines of 13, 19, 20 and 21: The concentration of imidazole was written in mM of lysis buffer.*

**7.** The authors perform a NiNTA purification step, and then follow it with a heat incubation step. However, in their purification table the specific activity decreases significantly after the heat incubation step. Normally the highest specific activity material is utilized for biochemical study. Why did the authors settle upon a lower specific activity material?

*In this study, we used a purification step using a Ni Sepharose column (NiNTA) and purified the protein 1147 by the presence of the histidine tag. We also used a heat shock step due to the temperature stability of the protein 1147, instead of Ni Sepharose (NiNTA) column. Our rationale for using this heat shock method as well as reporting and comparing it with Ni Sepharose (NiNTA) method was that in general, purification processes are time-consuming and costly, especially if protein production is required in the large scale (such as pilot and even industrial).*

*If we can reduce the time to purification and the number of steps, then we can increase the cost-efficiency. If this protein needs to be purified on an industrial scale, the use of a heat shock purification step can greatly save cost and time.*

*The use of heat shock reduced the enzyme activity by 10% in this study, but if the enzyme needs to be produced and purified on a larger scale, the net efficiency, cost reduction, time and number of purification steps can be justified.*

**8.** The authors quote the ProtParam web server reported instability index as "confirming" the protease structure is stable. Such analyses confirm nothing, they only suggest

*Given that protein 1147 is inherently a thermophilic bacterium, we used data from the online software Protparam to show that factors related to the thermal properties of a protein were also found in protease 1147. However, we decided to move Table 1 to the supplementary file.*

**9.** The authors state that ANS binding is related to exposure of hydrophobic patches. More generally, ANS binding is considered to indicate molten globule structure

*Thank you for the comment. We agree with the refree. A MG phase is observed. We provide further explanations in the following paragraph. We also mentioned this in the manuscript in green font (page 31 in discussion session).*

*Using ANS binding to protein investigated at 40 to 100 °C showed that fluorescence emission occurred at temperatures at which the hydrophobic regions of the protein were exposed to this solvent. Therefore, it was concluded that the compact structure of the enzyme 1147 is formed at 60 °C because due to inaccessibility of ANS to the internal domain and lack of binding. The maximum binding of ANS to the enzyme was at 100 °C, which could be due to the loss of protein folding and hydrophobic groups exposed to the solvent at this temperature. The maximum fluorescence emission from 60 °C to 100 °C was a blue-shift from 480 nm to 474 nm. The decrease of fluorescence that occurs as a result of the inability of the ANS binding to the hydrophobic region indicates that the enzyme maintains its structure from 40 to 90 °C.*

*The enzyme at pH 1and 2exhibited the highest fluorescence emission. When the ANS cannot bind to the hydrophobic region of the enzyme, fluorescence emission does not occur. The closer to the optimum pH of the enzyme, the lower the binding rate of ANS to the enzyme. Compared to the natural protein structure (pH 7), when the pH was reduced to 3, a slight blue shift (4 nm) was observed in maximum fluorescence emission, which may indicate the presence of intermediate state (MG). The intensity of the ANS fluorescence spectrum is consistent with the activity of protease 1147 in the presence of casein substrates.*

**10.** The authors report spectroscopic data as a function of temperature and pH. In principle, such data can be deconvoluted (e.g. with two-state, reversible denaturation models) to fit thermodynamic parameters of stability (i.e. DeltaG of unfolding, and so on). The authors however do not carry their analysis through to this point. In viewing the data presented, it is possible that unfolding is not two-state or cooperative (but this is speculation in the absence of any such analysis).

*It is assumed that irreversible denaturation of proteins is a two-step reaction:*

*N ↔ U→ I*

*N is the native protein form, U is the partially unfolded protein state and I is the irreversible inactivated protein form.*

*https://doi.org/10.1038/s41598-019-55587-9*

**11.** In general, a number of figures or tables are not so significant and could be put into supplemental data. In other words, the authors fill the report with figures and tables of questionable significance.

*We improved some of the Figures(Fig.s 1, 2, 3, 7), table 1 was moved to supplementary and the tables 1, 2, and 3 were updated.*

**Reviewer #3:**

The manuscript by Tarrahimofrad et al. describes the production and characterization of a thermostable protease. While the topic of the manuscript is interesting, and the enzyme described may find some commercial application, there are multiple issues that need to be addressed/clarified.

**1. Page 5, lines 21-22**

This sentence is confusing, as it suggests that the gene is thermostable and not the protein.

*The sentence is changed to “The goal of the present study was recombinant expression of a previously isolated thermostable protease gene (Coh01144)”.*

**2. Page 7, line 16**

Please add information on sequence identity and similarity between P. *horikoshii* protease and the protease 1147.

*This comparison is shown in Figure 2, where the amino acid sequence of Protease 1147 and P. horikoshii has been compared and the conserved sequences between these two proteins and several other similar proteins to Protease 1147 have been identified. (In Figure 2, part A; the first and the last sequence belong to protease 1147 and P. horikoshii*, respectively*).*

*The 3D superimposition between the native structure of Pyrococcus horikoshii chain A and predicted protease 1147 structure was shown in fig 3C.*

**3. Page 8, line 7**

The title of the paragraph should be rather “Protein-ligand biding – docking studies”.

*The title was changed to “Protein-ligand docking studies” and specified in blue font.*

**4. Page 10, line 4**

There is “bacterial colon” and it should read “bacterial colony”.

*Thanks for the comment. The typo was corrected.*

**5. Paragraph on recombinant protein purification**

In buffers’ formulations the imidazole concentration should be reported instead of milligrams quantities of the compound.

*Page 9, Lines of 16 snd 23; Page 10, Lines of 1 and 2: The concentration of imidazole was written in mM*

**6.** To reference various manuscripts the authors use “et al” and it should read “et al.”.

*Page 11, Line 6 and 20: Both cases were corrected in the manuscript.*

**7. Table 1** can be moved to the Supplementary Materials
Please add the unit for MW.
There is “Pi” and should be “pI”.

*The MW unit was added to the manuscript and Pi was also corrected and pI replaced. The table be moved to Supplementary.*

**8. Page 19, line 7**
Unit for the reported RMSD value is missing.
*Unit for RMSD value (nm) was added to the graph and text.*

**9.** Generally all tables are poorly looking and some work has to be done to make them more esthetically pleasing.

*The updated tables were uploaded.*

**10.** Please comment on the possible influence of the polyhistidine tag on the enzymatic activity of the protease.

*According to the bioinformatics analysis the amino acids of the active site of the enzyme 1147 are located in the middle of the protein sequence (Glu^77^, Cys^103^, His^104^, and Gly^105^). Since the histidin tag was incorporated at the C-terminal position of the enzyme, it has a logical distance that one can expects no interference. Furthermore the result of docking with Tween-20 and SDS did not show the establish of hydrogen bonding with the C-terminal his-tag*

*To be more assure about the possible influence of his-tag, we modeled the structure with and without his-tag and superimpose them. As it is shown in the figure R3, no structural changes are observable.*

*The long distance between the amino acids of active site and his-tag is shown in figure R3.*

Figure R2. The superimposition of 1147 with polyhistidine tag (Beige color) and 1147 without polyhistidine tag (cyan color). The polyhistidine tag residues showed in red color.

Figure R3. The apparent distance between the amino acids of the protease active site 1147 and the histidine tag. For clarity, the amino acids of the histidine tag are named.

**11.** Please provide information on the quaternary form of the protein. In the case of the protease from P. horikoshii only the oligomeric form of the enzyme was active.

*Protein 1147 after Blast-PDB showed the most similarity to P. horikoshii protein A chain. Therefore, modeling homology was performed based on the PDB structure of P. horikoshii protein. Although P. horikoshii has been reported to be active in its oligomeric form, Protease 1147 was able to function in its monomeric form. Identification and determination of monomer-to-decamer protein forms require the use of advanced crystallographic techniques such as X-Ray and NMR. These methods are accurate and efficient. However, it takes a lot of time and cost. At this point, we used the integration of software and a server to predict the quaternary form of Protease 1147. Initially using MODELLER software, modeling of protease 1147 on horikoshii, in addition to chain A, protease 1147 structure on chain B and chain C of protease horikoshii were also performed. This structure was represented by Chimera.*

*In addition to this step, we also predicted the fourth structure of protease 1147 based on the PDB structure of P. horikoshii protein in two forms of Asymmetric Unit and Biological Assembly 1 by the ZDOCK server. In this server, by selecting and introducing the binding site in all three chains, the protein structure was obtained in the Biological Assembly form and the protease 1147 quaternary form. The results related to the prediction of forth structure of protease 1147 are shown in figures R4-10.*

Figure R4. The Cohnella sp. A01 protease active domain in Asymmetric Unit form.

Figure R5. The crystal structure of intracellular protease from Pyrococcus Horikoshii PH1704 in Asymmetric Unit form.

Figure 6. The superimposition of Cohnella sp. A01 protease 1147 and Pyrococcus Horikoshii PH1704 in Asymmetric Unit form.

Figure 7. The Cohnella sp. A01 protease 1147 quaternary chematic.

Figure 8. The superimposition of Cohnella sp. A01 protease 1147 and Pyrococcus Horikoshii PH1704 quaternary chematic.

Figure 9. The crystal structure of intracellular protease from Pyrococcus Horikoshii PH1704 in Biological Assembly form.

Figure 10. The superimposition of Cohnella sp. A01 protease 1147 and Pyrococcus Horikoshii PH1704 in Biological Assembly form.

**12.** Please use “kcat” instead of “Kcat”.

*Thanks for the comment. The word K_cat_ was replaced with k_cat_ in the text.*

**13. Table 3**
Please correct the way how the errors are reported. For example, there is “91.81±2.7” and it should be “91.8±2.7”. Please check the error reported for SDS, as currently it is zero.

*Thanks for the comment. The data were corrected.*

**14. Table 4**

Please report the percentage values of the secondary structure without decimals.
How do these values compare with the percentage of the secondary structure calculated based on the protein model?

*We reported the percentage values of the secondary structure with one decimals*. *We used bioinformatics softwares to predict the secondary structure. However, in this study, we calculated, in real and in vitro terms, the extent of the protease1147 structure using Circular Dichroism. We have the secondary structure information after 40 ns, whereas for spectroscopic studies, the enzymatic assay conditions were maintained for 15 minutes, after which the secondary structures were read. Therefore, secondary structure information is not comparable.*

**15.** Please report KM and Vmax values together with their experimental errors.

*Thanks for the comment. We reported Km and Vmax values with their experimental errors.*
