# Supplementary material for: Structural and biochemical characterization of a novel thermophilic Coh01147 protease
Source: PLoS One. 2020 Jun 23;15(6):e0234958. doi: 10.1371/journal.pone.0234958 (PMC7310833; doi:10.1371/journal.pone.0234958)
Supplement: Attachment — Submitted filename: Review_Comments_PONE-D-19-30576R1.doc [file pone.0234958.s008.doc]

The revised version of the manuscript entitled “Structural and biochemical characterization of a novel thermophilic Coh01147 protease” (numbered: PONE-D-19-30576R1) has been well improved versus its original form, and it can be published in the journal PLOS ONE.

The above decision is based on that, generally, authors have responded carefully in most of the queries of authors, and were conformed to their constructive comments. On the other hand, and in my opinion, some responses of the authors to the comments of the reviewers are not satisfactory. Additionally, authors should take seriously into account more issues (see below) in their future publications.

In more details:

(a) Reviewer #2, comment #5: I think that the core of this question is related to the fact that authors did not used, as substrate, some relatively low molecular weight synthetic peptide specific for cysteine proteases (there are a plethora of them on the market), instead of casein (a protein). Authors’ responses “…enzyme 1147 acts as an active domain of protease function…”, along with “…We just performed Tween20 and SDS as ligand for molecular modeling and did not performed substrate….”, are confusing. Personally, I would strongly suggest to authors the use, in their future works, low molecular weight synthetic peptides, as proteases’ substrates; these latter offer to the experimenter much more information, especially in kinetic measurements (e.g. in the estimation of k_cat_, K_m_, k_cat_/K_m_, in pH- and temperature- profiles, etc).

(b) Reviewer #2, comment #10: It seems that more likely authors misunderstood the reviewer’s query and comments; in the cited address “https://doi.org/10.1038/s41598-019-55587-9” is referred a similar speculation. In my opinion these comments of Reviewer #2 are correct.

(c) Additional: Authors based some of their results on false and/or old-fashion, and statistically erroneous, treatments of their experimental kinetic data (original and revised versions of the manuscript).

(I) Authors estimated the M.M. parameters (k_cat_ or V_max_, and K_m_) using the Lineweaver-Burk plot, i.e. the statistically most erroneous method; it has been rejected since 1961 (G.N. Wilkinson, The Biochemical journal, **80**, 1961, 324–332). Nowadays, are easily available numerous statistically robust non-linear computer programs/algorithms, based also on non-parametric statistics, which are the specific ones for data from enzyme kinetic experiments. It is too pity those authors, who handle more complicated computer subroutines, to ignore and/or to underestimate important issues; this sounds unpleasant for the reputation of the authors!

(II) Figure 9: as ordinate in all four diagrams is the “Relative activity %”, which is meaningless and worthless according to the Current IUBMB recommendations on enzyme nomenclature and kinetics (A. Cornish-Bowden, Perspectives in Science **1**, 2014, 74-87). The accepted meaningful entities, which should be used as ordinates in similar diagrams, are the M.M parameters k_cat_ or V_max,_ K_m_, k_cat_/K_m_ or V_max_/K_m_. Furthermore, the pH-profiles of the aforementioned entities (Fig 9C) should be fitted using proper equations (e.g. ref. E.M. Papamichael et al, “Enzyme Kinetics and Modeling of Enzymatic Systems”, *in* *ADVANCES IN ENZYME TECHNOLOGY*, 1^st^ Edition, p. 83/Eqs 3.11-3.14, and ref. A. Foukis et al, Bioresource Technology **123**, 2012, 214–220/Eqs 1 and 1a), and estimate all important pKa-values. Likewise, the temperature profiles (Fig 9A) in all cases should be depicted by using the absolute scale as abscissa. Subsequently, the experimental data should be successively fitted by variants of the Arrhenius’ and Eyring equations (e.g. ref. A. Foukis et al, Bioresource Technology **123**, 2012, 214–220/Eqs 2 and 3). In the case of the absolute temperature profiles, the activation thermodynamic parameters *Δ*G^‡^, Δ*H*^‡^ and Δ*S*^‡^ could be estimated easily and more precisely (e.g. ref. E.M. Papamichael et al, “Enzyme Kinetics and Modeling of Enzymatic Systems”, *in* *ADVANCES IN ENZYME TECHNOLOGY*, 1^st^ Edition, p. 83/Eqs 3.17-3.18/3.15-3.16).
