# Supplementary material for: Structural and biochemical characterization of a novel thermophilic Coh01147 protease
Source: PLoS One. 2020 Jun 23;15(6):e0234958. doi: 10.1371/journal.pone.0234958 (PMC7310833; doi:10.1371/journal.pone.0234958)
Supplement: Attachment — Submitted filename: Response to Reviewers.docx [file pone.0234958.s009.docx]

**Journal:**	*PLOS ONE*

**Manuscript #:**	*PONE-D-19-30576R1*

**Title of Paper:** *Structural and Biochemical Characterization of a Novel Thermophilic*

*Coh01147 Protease*

**Authors:**	*Hossein Tarrahimofrad, Amir Meimandipour, Sareh Arjmand, Mohammadtaghi Beigi Nassiri, Ehsan Jahangirian, Hossein Tavana, Javad Zamani, Somayyeh Rahimnahal, Saeed Aminzadeh*

**Dear Dr. Paulo Lee Ho**

We appreciate the time and efforts by the editor and referees in reviewing this manuscript. We have addressed all issues indicated in the review report carefully and believed that the revised.

On your recommendation, we have recorded a number of our own materials and methods in <https://www.protocols.io/> and put the DOI of each in the manuscript.

We while revisied our submission, uploaded our all figure files to the Preflight Analysis and Conversion Engine (PACE) digital diagnostic tool, <https://pacev2.apexcovantage.com/>

**Yours sincerely**

**Saeed Aminzadeh**

We appreciate the reviewers who took the time to read the manuscript and helped us improve the manuscript with their comments. We have carefully applied the valuable comments of the esteemed reviewers in the manuscript.

**Reviewer #3**: Please check the unit for K_M_. It reads "mg/ml-1" and it should be "mg/ml". All previously listed issues were addressed.

*The K_m_ unit was corrected and expressed as “*mM”*. This unit was obtained using Prism GraphPad V.8 software and addressed in all previously listed issues:*

***Page 2, line 14.***

***Page 23, line 4 and 5.***

***Page 24, table 2.***

***Supplementary, S1 file, page 7, table D.***

**Reviewer #4:** The revised version of the manuscript entitled “Structural and biochemical characterization of a novel thermophilic Coh01147 protease” (numbered: PONE-D-19-30576R1) has been well improved versus its original form, and it can be published in the journal PLOS ONE.

The above decision is based on that, generally, authors have responded carefully in most of the queries of authors, and were conformed to their constructive comments. On the other hand, and in my opinion, some responses of the authors to the comments of the reviewers are not satisfactory. Additionally, authors should take seriously into account more issues (see below) in their future publications.

In more details:

**(a) Reviewer #2, comment #5:** I think that the core of this question is related to the fact that authors did not used, as substrate, some relatively low molecular weight synthetic peptide specific for cysteine proteases (there are a plethora of them on the market), instead of casein (a protein). Authors’ responses “…enzyme 1147 acts as an active domain of protease function…”, along with “…We just performed Tween20 and SDS as ligand for molecular modeling and did not performed substrate….”, are confusing. Personally, I would strongly suggest to authors the use, in their future works, low molecular weight synthetic peptides, as proteases’ substrates; these latter offer to the experimenter much more information, especially in kinetic measurements (e.g. in the estimation of kcat, Km, kcat/Km, in pH- and temperature- profiles, etc).

*We thank the referee for the explanation. You are absolutely right. As you said, unfortunately, we misunderstood the referee's intent and answered it based on a misunderstanding of the question.*

*We also thank you for your advice. Understanding your advice, we decided to use small synthetic substrates instead of casein in our future studies.*

**(b) Reviewer #2**, comment #10: It seems that more likely authors misunderstood the reviewer’s query and comments; in the cited address “https://doi.org/10.1038/s41598-019-55587-9” is referred a similar speculation. In my opinion these comments of Reviewer #2 are correct.

*Proteins in monomer mode are considered as two states. It is assumed that irreversible denaturation of proteins is a two-step reaction:*

*N ↔ U→ I*

*N is the protein native state, U is the reversibly and partially unfolded enzyme form and I is the enzyme irreversible inactivated state. The transition state (Tn^*^) which is formed between N and U is determining the irreversible thermodynamic parameters [**1**,* *2**].*

*Considering that we have considered the enzyme form monomer in all stages of bioinformatics and laboratory experiments by default. Therefore, when we have the monomer form of the protein, the protein can be considered as two states.*

*In addition, in the laboratory, we used PAGE with a non-regenerative form when examining the activity of the enzyme with a zymogram test. If the enzyme is a monomer, the protein will be in its true size, but if the protein is in the form of a dimer or multimeter, the protein will be in the area above its molecular weight. The 1147 protease was found in the non-regenerative PAGE of the zyogram, in front of its molecular weight about of 18.5 kDa (Fig 8B, in manuscript).*

*The above allows us to consider the enzyme protease 1147 as a monomer protein.*

*ΔG^#^ is the value usually shows the protein denaturation reaction. ΔH^#^ and ΔS^#^ are determining respectively the heat and entropy alteration in conversion reaction of native to the transition state. To verify this statement, we calculated the thermodynamic parameter of the irreversible deactivation energy and considered it as a function of the thermodynamic performance (ΔG^#^ of unfolding) of the protease 1147 instade of deconvolve CD data with two-state, irreversible denaturation models.*

**(c) Additional**: Authors based some of their results on false and/or old-fashion, and statistically erroneous, treatments of their experimental kinetic data (original and revised versions of the manuscript).

*We recalculated the kinetic data using the software and adjusted the other results based on the new calculations.*

**(I)** Authors estimated the M.M. parameters (kcat or Vmax, and Km) using the Lineweaver-Burk plot, i.e. the statistically most erroneous method; it has been rejected since 1961 (G.N. Wilkinson, The Biochemical journal, 80, 1961, 324–332). Nowadays, are easily available numerous statistically robust non-linear computer programs/algorithms, based also on non-parametric statistics, which are the specific ones for data from enzyme kinetic experiments. It is too pity those authors, who handle more complicated computer subroutines, to ignore and/or to underestimate important issues; this sounds unpleasant for the reputation of the authors!

*We read the reference introduced by you and we follow your advice. We recalculated all the enzyme kinetic arguments based on statistical nonlinear computer programs and algorithms using prism software, and we put them in the manuscript and corrected all the results based on the new M.M parameters.*

***Page 11, line 16 and 17.***

***Page 23, line 4 and 5.***

***Supplementary, S4 Fig.***

(II) Figure 9: as ordinate in all four diagrams is the “Relative activity %”, which is meaningless and worthless according to the Current IUBMB recommendations on enzyme nomenclature and kinetics (A. Cornish-Bowden, Perspectives in Science 1, 2014, 74-87). The accepted meaningful entities, which should be used as ordinates in similar diagrams, are the M.M parameters kcat or Vmax, Km, k_cat_/Km or Vmax/K_m_.

*All diagrams related activity were corrected and reported based on the V_max_ parameter:*

***Figure 9 A, B, C & D.***

Furthermore, the pH-profiles of the aforementioned entities (Fig 9C) should be fitted using proper equations (**e.g. ref. E.M. Papamichael et al, “Enzyme Kinetics and Modeling of Enzymatic Systems”, in ADVANCES IN ENZYME TECHNOLOGY, 1st Edition, p. 83/Eqs 3.11-3.14,** and **ref. A. Foukis et al, Bioresource Technology 123, 2012, 214–220/Eqs 1 and 1a**), and estimate all-important pKa-values. Likewise, the temperature profiles (Fig 9A) in all cases should be depicted by using the absolute scale as abscissa.Subsequently, the experimental data should be successively fitted by variants of the Arrhenius’ and Eyring equations (**e.g. ref. A. Foukis et al, Bioresource Technology 123, 2012, 214–220/Eqs 2 and 3**).

*Thank you very much for your guidance. We've read the top-level references you've introduced, and we've come up with some very important new ones.*

*We are pleased to announce that we are currently studying the 1147 Protease Crystallography, and we plan to use the tips and equations you have introduced in our next article, along with information on crystallography and low molecular weight synthetic substrate.*

*Also, as the amount of content presented in our current study increases, we plan to use and report your proposed equations in our next study.*

In the case of the absolute temperature profiles, the activation thermodynamic parameters ΔG‡, ΔH‡ and ΔS‡ could be estimated easily and more precisely (**e.g. ref. E.M. Papamichael et al, “Enzyme Kinetics and Modeling of Enzymatic Systems”, in ADVANCES IN ENZYME TECHNOLOGY, 1st Edition, p. 83/Eqs 3.17-3.18/3.15-3.16**).

*Thank you very much. We used the appropriate equation (Eyring) introduced in your book and calculated the data for estimating the energy activation of the enzyme and other thermodynamic parameters related to the conversion of the enzyme to the product. Based on this, we brought the relevant data in the materials and methods section, the results, as well as the discussion. In this way, this data was updated in two tables and one section added in Photo 9.* *After completing and replying to your comment, we have identified the all items required in the manuscript:*

*Material and methods:* ***Page 13, line 17*** ***to*** ***page 16, line 12.***

*Resultes:* ***Page 23, line 19 to page 25, line 2.***

*Discussion:* ***Page 33, line 14 to page 34, line 23.***

**Refrences:**

1.	Babashpour S, Aminzadeh S, Farrokhi N, Karkhane A, Haghbeen K. Characterization of a Chitinase (Chit62) from Serratia marcescens B4A and Its Efficacy as a Bioshield Against Plant Fungal Pathogens. Biochemical Genetics. 2012;50(9):722-35. doi: 10.1007/s10528-012-9515-3.

2.	Mosallatpour S, Aminzadeh S, Shamsara M, Hajihosseini R. Novel halo- and thermo-tolerant Cohnella sp. A01 L-glutaminase: heterologous expression and biochemical characterization. Scientific Reports. 2019;9(1):19062. doi: 10.1038/s41598-019-55587-9.
